# Wor1‐regulated ferroxidases contribute to pigment formation in opaque cells of *Candida* *albicans*

**DOI:** 10.1002/2211-5463.13070

**Published:** 2021-02-18

**Authors:** Baodi Dai, Yinxing Xu, Ning Gao, Jiangye Chen

**Affiliations:** ^1^ State Key Laboratory of Molecular Biology Shanghai Institute of Biochemistry and Cell Biology Center for Excellence in Molecular Cell Science Chinese Academy of Sciences Shanghai China

**Keywords:** *Candida albicans*, opaque cells, Wor1, ferroxidases, melanin

## Abstract

*Candida albicans* is a harmless commensal resident in the human gut and a prevalent opportunistic pathogen. A key part of its commensalism and pathogenesis is its ability to switch between different morphological forms, including white‐to‐opaque switching. The Wor1 protein was previously identified as a master regulator of white‐to‐opaque switching in mating type locus (*MTL*) homozygous cells. The mechanisms by which the dark color of the opaque colonies is controlled and the pimpled surface of opaque cells is formed remain unknown. *Candida albicans* produces melanin pigment *in vitro* and during infection. However, the molecular mechanism underlying the regulation of melanin production is unclear. In this study, we demonstrated that ferroxidases (Fets) function as pigment multicopper oxidases and regulate the production of dark‐pigmented melanin in opaque cells. The *FET* genes presented distinct regulation patterns in response to different extracellular stimuli. In YPD (1% yeast extract, 2% peptone and 2% dextrose)‐rich medium, four of the five *FET* genes were up‐regulated by Wor1, especially at the human body temperature of 37 °C. In minimal medium with low ammonium concentrations, all five *FET* genes were up‐regulated by Wor1. However, at high ammonium concentrations, some *FET* genes were down‐regulated by Wor1. Wor1‐up‐regulated Fets contributed to dark pigment formation in opaque colonies, but not to the elongated shape of these opaque cells. Increased melanin externalization was associated with the pimpled surface of the opaque cells. Melanized *C. albicans* cells were more resistant to fungal clearance. Deletion of the five *FET* genes completely blocked melanin production in opaque cells and resulted in the generation of white elongated ‘opaque’ cells. In addition, the up‐regulated Fets are important for defense against oxidant attacks. The functional diversity of Fets may reflect the multiple strategies of *C. albicans* to rapidly adapt to diverse host niches.

## Graphical abstract

In *Candida albicans* opaque cells, the master regulator Wor1 up‐regulates Fets, which function as pigment multicopper oxidases and promote the production of dark‐pigmented melanin. Increased melanin externalization is associated with the pimpled surface and dark color of opaque cells. The melanized *C. albicans* cells were more resistant to fungal clearance, including bone marrow‐derived macrophage phagocytosis and oxidative killing.

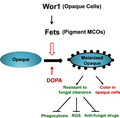

**Abbreviations**
AMB: amphotericin B
BMDM: bone marrow‐derived macrophage
CFU: colony‐forming unit
CnLac1: *Cryptococcus neoformans* Lac1
DHN: 1, 8‐dihydroxynaphthalene
Fets: ferroxidases
GI: gastrointestinal
GlcNAc: *N*‐acetylglucosamine
GMM: glucose minimal medium
GMM‐LA: glucose minimal medium with low ammonium
GUT: gastrointestinally induced transition
HsCp: *Homo sapiens* ceruloplasmin
KO: knockout
l‐DOPA: l‐3,4‐dihydroxyphenylalanine
MCO: multicopper oxidase
*MTL*: mating type locus
OD: optical density
PM: plasma membrane
qPCR: quantitative PCR
SCD: synthetic complete medium with glucose
SD: standard deviation
SEM: scanning electron microscopy
TEM: transmission electron microscopy
WT: wild‐type
YPD: 1% yeast extract, 2% peptone and 2% dextrose

*Candida albicans* is a harmless commensal resident in the human gut and a prevalent opportunistic pathogen [1, 2, 3]. Important to its commensalism and pathogenesis is its ability to switch between different morphological forms, including white‐to‐opaque switch and white‐to‐gastrointestinally induced transition (GUT) [3, 4, 5, 6, 7]. The white and opaque phenotypes have different colonial morphologies in terms of colony size, shape and color. White cells form hemispherical and white‐colored colonies, whereas opaque cells form much larger, flatter and opaque or dark‐colored colonies. Opaque cells are elongated with a pimpled surface, but the mechanisms that control the dark color of opaque colonies and the pimpled surface of opaque cells remain unknown. White cells are more virulent than opaque cells in systemic infections, whereas the virulence of opaque cells seems to increase during cutaneous infection [8, 9]. Opaque cells are significantly less susceptible to phagocytosis by *Drosophila* and mouse phagocytes than white cells [10]. GUT cells are elongated in shape with a smooth surface. GUT cells showed enhanced commensal fitness when passing through the mammalian gastrointestinal (GI) tract; however, they attenuated virulence in the bloodstream [3, 11].

Wor1 was previously identified as a master regulator of white‐to‐opaque switching in mating type locus (*MTL*) homozygous cells [12, 13, 14]. Chromatin immunoprecipitation assay showed that it binds to more than 100 target promoters and its own upstream region [15]. Wor1 is expressed when passing through the mammalian gut, thus triggering white‐to‐GUT switching in *MTL* ***a*** */α* heterozygous cells and promoting *C. albicans* commensalism [3]. Wor1 overexpression also increases the adhesion of *C. albicans* cells to the mouse gut mucosa [16].

Melanin is usually described as one of the most common natural pigments and is broadly produced by various organisms [17, 18]. Melanins are negatively charged hydrophobic macromolecules, which are composed of polymerized phenolic and/or indolic compounds, and referred to as ‘fungal armor’ because of the ability of the polymer to protect microorganisms against a broad spectrum of toxic insults. Several types of melanin are known to exist in the fungal kingdom, most of which are derived from 1, 8‐dihydroxynaphthalene (DHN), known as DHN‐melanin, and the others are derived from l‐3,4‐dihydroxyphenylalanine (l‐DOPA), known as DOPA‐melanin [19]. DHN‐melanin is usually synthesized by filamentous fungi, such as *Aspergillus* spp., through a polyketide synthase pathway, by using endogenous substrates, including acetyl‐CoA and malonyl‐CoA. After a series of hydrolysis, reduction, dehydration and polymerization, DHN‐melanin is formed. In contrast, DOPA‐melanin is produced via a polyphenoloxidase (a laccase), which catalyzes a one‐step oxidation of dihydroxyphenols to quinone intermediates, which subsequently auto‐oxidize to form melanin [17, 18]. *Cryptococcus neoformans* produces pigments from many aminophenol and diaminobenzene compounds *in vitro* [20]. During central nervous system infection *in vivo*, *C. neoformans* synthesizes melanin from neurotransmitters, such as dopamine, norepinephrine and epinephrine as substrates [21]. *C. neoformans* cannot generate melanin without an exogenous substrate, and the type of pigment synthesized varies depending on the chemical structure of the substrate added to the medium [18]. *C. neoformans* melanin is catalyzed by laccases and encoded by two tandem localized genes, *CNLAC1* and *CNLAC2* [22, 23], and *CNLAC1* plays a dominant role in melanin production [24, 25].

Laccases constitute the largest subfamily of multicopper oxidases (MCOs) and are widely distributed in fungi, higher plants, bacteria and insects. MCOs consist of four enzyme families: laccases, ascorbate oxidases, ferroxidases (Fets) and ceruloplasmin [26]. Most MCOs can use a wide variety of aromatic phenols and amines as reducing substrates. Laccases and Fets share some MCO‐specific patterns and signature sequences (https://lcced.biocatnet.de/) [27]. Fets can oxidize laccase substrates in addition to ferrous iron at a lower catalytic efficiency. Laccases also can oxidize ferrous iron other than phenolic substrates at a lower rate [28]. In the *C. albicans* genome (http://www.candidagenome.org), there are five predicted MCOs, which were previously reported as Fets [29]. Interestingly, the five Fets showed distinct iron‐dependent regulation patterns, and two of them (Fet34 and Fet99) were significantly up‐regulated by low iron [29]. Whether the five Fets can oxidize laccase substrates and produce pigments in *C. albicans* remains to be investigated.

*C. albicans* can produce melanin pigment *in vitro* and during infection [30]. Melanin externalization also has been observed in *C. albicans* [31]. In this study, we proved that the previously reported Fets could function as pigment MCOs necessary for melanin pigment production. We found that all the *FET* genes presented distinct regulation patterns in response to different extracellular stimuli. The Wor1‐up‐regulated Fets contributed to dark pigment formation in opaque cells, but not to the elongated shape of opaque cells. To the best of our knowledge, this is the first study to link the dark color of opaque cells to melanin pigment production and the pimpled surface of opaque cells to melanin externalization, thus revealing the functional diversity of Fets in *C. albicans*.

## Materials and methods

### Strains and growth conditions

The *C. albicans* strains used in this study are listed in Table 1. *C. albicans* strains were routinely grown at 22 °C in YPD (1% yeast extract, 2% peptone and 2% dextrose). YPS (YP + 2% sorbose) was used to isolate *MTL* ***a/a*** or *MTLα/α* strains. Transformants were selected on synthetic complete medium with glucose (SCD; 2% dextrose, 0.17% yeast nitrogen base without amino acids and ammonium sulfate, 0.5% ammonium sulfate and auxotrophic supplements). Cells were cultured in minimal medium (15.0 mm glucose, 10.0 mm MgSO_4_, 29.4 mm KH_2_PO_4_, 13.0 mm glycine, 3.0 m vitamin B_1_, pH 5.5) with l‐DOPA and *N*‐acetylglucosamine (GlcNAc) for melanin production. Glucose minimal medium (GMM; 2% w/v glucose, 0.17% w/v yeast nitrogen base without amino acids and ammonium sulfate, and 0.5% w/v ammonium sulfate) and GMM with low ammonium (GMM‐LA; 2% w/v glucose, 0.17% w/v yeast nitrogen base without amino acids and ammonium sulfate, and 0.025 mg·L^−1^ ammonium sulfate) were used to test the effects of iron on *FET* expression and the impact of Wor1 on *FET* expression. To prepare the minimal medium containing different concentrations of iron, we first depleted GMM or GMM‐LA of iron by adding 1 mm ferrozine and 10 mm ascorbic acid, followed by the addition of different amounts of FeCl_3_.

**Table 1:** Strains used in this study.

Strain	Parent	Mating type	Description	Genotype	Reference
SC5314		***a*** */α*		WT	[65]
SC5314 ***a/a***	SC5314	***a/a***		WT	This study
CAI4	CAF2‐1	***a*** */α*	*ura3*::λ *imm434/ura3*::λ *imm434*	WT	[36]
BWP17	RM1000	***a*** */α*	*ura3*::λ *imm434/ura3*::λ *imm434 his1*::*hisG/his1*::*hisG arg4*::*hisG/arg4*::*hisG*	WT	[33]
JYC1	BWP17	***a/a***	*ura3::λ imm434/ura3::λ imm434 his1::hisG/his1::hisG arg4::hisG/arg4::hisG*	WT	[66]
JYC5	CAI4	***a/a***	*ura3*::λ *imm434/ura3*::λ *imm434*	WT	[66]
CDB1	BWP17	***a*** */α*	*ura3::λ imm434/ura3::λ imm434 his1::hisG/his1::hisG arg4::hisG/arg4::hisG fet3::ARG4/fet3::HIS1*	*fet3Δ/Δ*	This study
CDB2	CDB1	*α/α*	*ura3::λ imm434/ura3::λ imm434 his1::hisG/his1::hisG arg4::hisG/arg4::hisG fet3::ARG4/fet3::HIS1*	*fet3Δ/Δ*	This study
CDB3	BWP17	***a*** */α*	*ura3::λ imm434/ura3::λ imm434 his1::hisG/his1::hisG arg4::hisG/arg4::hisG fet31::ARG4/fet31::HIS1*	*fet31Δ/Δ*	This study
CDB4	CDB3	***a/a***	*ura3::λ imm434/ura3::λ imm434 his1::hisG/his1::hisG arg4::hisG/arg4::hisG fet31::ARG4/fet31::HIS1*	*fet31Δ/Δ*	This study
NKX1	BWP17	***a*** */α*	*ura3::λ imm434/ura3::λ imm434 his1::hisG/his1::hisG arg4::hisG/arg4::hisG fet33::ARG4/fet33::URA3‐dpl200*	*fet33Δ/Δ*	[34]
CDB5	NKX1	***a/a***	*ura3::λ imm434/ura3::λ imm434 his1::hisG/his1::hisG arg4::hisG/arg4::hisG fet33::ARG4/fet33::URA3‐dpl200*	*fet33Δ/Δ*	This study
NKX5	BWP17	***a*** */α*	*ura3::λ imm434/ura3::λ imm434 his1::hisG/his1::hisG arg4::hisG/arg4::hisG fet34::ARG4/fet34::URA3‐dpl200*	*fet34Δ/Δ*	[34]
CDB6	NKX5	***a/a***	*ura3::λ imm434/ura3::λ imm434 his1::hisG/his1::hisG arg4::hisG/arg4::hisG fet34::ARG4/fet34::URA3‐dpl200*	*fet34Δ/Δ*	This study
CDB7	JYC1	***a/a***	*ura3::λ imm434/ura3::λ imm434 his1::hisG/his1::hisG arg4::hisG/arg4::hisG fet99::ARG4/fet99::HIS1*	*fet99Δ/Δ*	This study
SN148		***a*** */α*	*ura3::λ imm434/ura3::λ imm434 iro1::λ imm434/iro1::λ imm434 his1::hisG/his1::hisG arg4::hisG/arg4::hisG leu2::hisG/leu2::hisG*	WT	[35]
CDB8	SN148	***a*** */α*	*ura3::λ imm434/ura3::λ imm434 iro1::λ imm434/iro1::λ imm434 his1::hisG/his1::hisG arg4::hisG/arg4::hisG leu2::hisG/leu2::hisG (fet31‐fet99‐fet3‐fet34)::HIS1/(fet31‐fet99‐fet3‐fet34) ::LEU2*	*4fetsΔ/Δ*	This study
CDB9	CDB8	***a/a***	*ura3::λ imm434/ura3::λ imm434 iro1::λ imm434/iro1::λ imm434 his1::hisG/his1::hisG arg4::hisG/arg4::hisG leu2::hisG/leu2::hisG (fet31‐fet99‐fet3‐fet34)::HIS1/(fet31‐fet99‐fet3‐fet34) ::LEU2*	*4fetsΔ/Δ*	This study
YZM119		***a*** */α*	*ura3::λ imm434/ura3::λ imm434 his1::hisG/his1::hisG arg4::hisG/arg4::hisG (fet31‐fet99‐fet3‐fet34)::FRT/(fet31‐fet99‐fet3‐fet34)::ARG4 fet33::URA3/fet33::HIS1*	*5fetsΔ/Δ*	[29]
CDB10	YZM119	***a/a***	*ura3::λ imm434/ura3::λ imm434 his1::hisG/his1::hisG arg4::hisG/arg4::hisG (fet31‐fet99‐fet3‐fet34)::FRT/(fet31‐fet99‐fet3‐fet34)::ARG4 fet33::URA3/fet33::HIS1*	*5fetsΔ/Δ*	This study
YZM120		***a*** */α*	*ura3::λ imm434/ura3::λ imm434 his1::hisG/his1::hisG arg4::hisG/arg4::hisG (fet31‐fet99‐fet3‐fet34)::FRT/(fet31‐fet99‐fet3‐fet34)::ARG4 fet33::FRT/fet33::HIS1*	*5fetsΔ/Δ*	[29]
CDB11	YZM120	***a/a***	*ura3::λ imm434/ura3::λ imm434 his1::hisG/his1::hisG arg4::hisG/arg4::hisG (fet31‐fet99‐fet3‐fet34)::FRT/(fet31‐fet99‐fet3‐fet34)::ARG4 fet33::FRT/fet33::HIS1*	*5fetsΔ/Δ*	This study
CHY477	CAI4	*α/α*	*ura3*::λ *imm434/ura3*::λ *imm434 mtla1::HisG/MTLα ade2::HisG URA3 HisG/ade2::HisG*	WT	[43]

### Plasmid and strain construction

All plasmids used in this study are listed in Table 2. DNA sequences of *C. albicans* genes were obtained from the Candida Genome Database (http://www.candidagenome.org). SC5314 genomic DNA was used as a template for all PCR amplifications of *C. albicans* genes. The plasmid pBA1‐FET34, for the expression of *FET34* under the control of the *ADH1* promoter in *C. albicans*, was constructed by inserting a PCR fragment containing *FET34* into the BglI‐KpnI site of pBA1. The plasmid pFET33‐knockout (KO) was constructed for the disruption of *FET33* carrying the *CaURA3* marker. The disrupting vectors pCPC9/48/49/50 carrying *CaURA3*, *CmLEU2*, *CdHIS1* and *CdARG4* markers were used as templates to delete target genes by PCR‐based homologous recombination, as previously described [32]. LoxP‐*CmLEU2*‐LoxP (PLP), LoxP‐*CdHIS1*‐LoxP (PHP) and LoxP‐*CdARG4*‐LoxP (PAP) were assembled with 5′ and 3′ fragments of target genes by the fusion PCR method. The BWP17 strain [33] was used to construct a single gene mutant *fet31/fet31Δ/Δ*, *fet99/fet99Δ/Δ* or *fet3/fet3Δ/Δ*. The *fet33/fet33Δ/Δ* and *fet34/fet34Δ/Δ* mutants were gifts from M. Li [34]. The *MTL* ***a*** */α* *4fetsΔ/Δ* (*FET31*‐*FET99*‐*FET3*‐*FET34*) mutant was generated from the SN148 strain [35] by PCR‐based homologous recombination with *CmLEU2* and *CdHIS1* markers and then streaked onto YPS for isolation of *MTL* ***a***/***a*** *4fetsΔ/Δ* strains. The two copies of *FET33* were disrupted by the ‘URA‐BLAST’ method [36]. PstI‐digested pFET33‐KO was transformed into the *MTL* ***a/a*** *4fetsΔ/Δ* (CBD9) mutant to generate the *5fetsΔ/Δ* mutant (CBD12). All disruptions were confirmed by Southern blotting and PCR analysis (Fig. S1). The primers used for PCR amplification are listed in Table 3.

**Table 2:** Plasmids used in this study.

Plasmid	Description	Reference
pBA1	*C. albicans ADH1* promoter in pBES116, *URA3* for integration at *ADE2* locus	[38]
pPR671	*C. albicans ACT1* promoter, 13Myc‐FLAG, *HIS1* for integration at *RP10* locus	[38]
pACT1‐WOR1	3.0 kb *WOR1* coding sequence in pACT1, from *ACT1* promoter, *URA3* for integration at *ADE2* locus	[12]
pBA1‐FET34	1.9 kb *FET34* coding sequence in pBA1, from *ADH1* promoter, *URA3* for integration at *ADE2* locus	This study
pBES116	*ADE2‐URA3‐ADE2* AscI fragment in pBluescript II KS(1)	[67]
pCPC9	Vectors pCPC9 carrying *CaURA3* marker amplified from pBES116	[32]
pCPC48	Vectors pCPC48 carrying *CmLEU2* marker amplified from pBES116	[32]
pCPC49	Vectors pCPC49 with *CdHIS1* marker amplified from pBES116	[32]
pCPC50	Vectors pCPC50 with *CdARG4* marker amplified from pBES116	[32]
pCUB6	Replacement of *S. cerevisiae URA3* by *C. albicans URA3* in pNKY50	[36]
pFET33‐KO	0.5‐ and 0.5‐kb KO fragments of *C. albicans FET33* and *hisG‐URA3‐hisG* in pBluescript SK(+)	This study

**Table 3:** The primers used in this study. F, forward primer (5'→3'); R, reverse primer (5'→3').

Primer name	Sequence (5ʹ→3ʹ)	Purpose and features
FET3‐F	CAGTATGGAGACGGTATGAG	qPCR primer for *FET3*
FET3‐R	TGTCGGATTAAATCTGGTTA	
FET31‐F	GATTTCCCATACGACTACGA	qPCR primer for *FET31*
FET31‐R	TTTACCTGGTTCAACTTTCC	
FET33‐F	GTGGTCGAACCATACGAAAC	qPCR primer for *FET33*
FET33‐R	TGTAAATCCAGTGGCAAGAA	
FET34‐F	GATGGTCCTGAAATGGTCAC	qPCR primer for *FET34*
FET34‐R	CAACATCCTCGTCATAATCG	
FET99‐F	AAAGTGCTCCATTGCCTGAT	qPCR primer for *FET99*
FET99‐R	CTCCGTTGCCTAAATTGTCC	
FTR1‐F	TCCAGCCACCTCTTTCCC	qPCR primer for *FTR1*
FTR1‐R	GAACAAACCAGCAGCAATCA	
FTR2‐F	GTGATGCGGCTGAGAATG	qPCR primer for *FTR2*
FTR2‐R	GGAAATGACAGAACCGTAAGTG	
FTH1‐F	CCCAACTGAATCTGATGACCT	qPCR primer for *FTH1*
FTH1‐R	GATAGCACCAATTATTAGACAAACC	
FTH2‐F	GAAACAGCTTCACCGCCAGTC	qPCR primer for *FTH2*
FTH2‐R	AACCCATAGAAATCCCACAAA	
WOR1‐F	CATGGTCCCCATCAAGAATC	qPCR primer for *WOR1*
WOR1‐R	GTCGTCGGGACCAAATTTAC	
ACT1‐F	GCTTTTGGTGTTTGACGAGTTTCT	qPCR primer for *ACT1*
ACT1‐R	GTGAGCCGGGAAATCTGTATAGTC	
FET3‐F1	TGGTTGGGTAGACGCAAATCCTGATGGGGTTTATCCCCG TGCCACCTGACGTCTAAGAA	KO *FET3* by PCR method
FET3‐R1	GTTCAAAACAACTGCTTCTTCTTCGTCCTCATTCTCTGC GGCCTTTTGCTCACATGTTC	
FET3‐F2	TTGATAGCTGCTGAAACTCACACATGGTATTTCAAAACTGGTTGGGTAGACGCAAATCC	
FET3‐R2	TGTGGCTTAGAGTTGCTGTCTGAAGAAGATGAATTCTGGTTCAAAACAACTGCTTCTTC	
FET3‐F3	CGACGCTTTGATTATTTC	
FET3‐R3	TTTGGTGATGGTGGTTTA	
FET31‐F1	GACCAATGATTGGGTTTAACGATAGTTGGCCGTTACCTATGCCACCTGACGTCTAAGAA	KO *FET31* by PCR method
FET31‐R1	ACCATAGTAGGCAATAGTGGCTAAACCTAAGACACCGGC GGCCTTTTGCTCACATGTTC	
FET31‐F2	CTGGTTGGGTGAATGCTAACCCAGATGGTGGATTTGAAA GACCAATGATTGGGTTTAAC	
FET31‐R2	TCTAGCAACTCTTTCTTCAACATCGGCAATGTCATTCATACCATAGTAGGCAATAGTGG	
FET31‐F3	CTTTGTCCATTTTCATTGGAGC	
FET31‐R3	CGGGATACGAATGGGGGGGGT	
FET33‐F1	TGCAAGATCTCTGCAGTAATGATATTACAACATTTGTTATAAATGG ATCAGC	KO *FET33* by URA‐BLAST method
FET33‐R1	TGCAAGATCTCCACCGCCACGCTCGCCACCTTCGCCCCATCGTCTTGTTTGAGATTGTT	
FET33‐F2	CGATGGATCCTACAATAAATGACTGCATTTAATAAACAATAGTAGTTGAT	
FET33‐R2	CGATGCATGCCTGCAGTTCATTAGCAGCAACACCTTTTTCAAGTAAACTGAACCCC	
FET99‐F1	TGGTTTAAACACATCTGAAAGATACAAGAACATTCTGGCTGCCACCTGACGTCTAAGAA	KO *FET99* by PCR method
FET99‐R1	ACACATATTTCTTCAAAAACCTCATTTAAATCCAAGATGGGCCTTTTGCTCACATGTTC	
FET99‐F2	CTGTTCTGCTCCCAATATTGAACACAACAGTATCGCTTTGGTTTAAACACATCTGAAAG	
FET99‐R2	ATAAACTCTTTTTCATACCTGGGTTATCTTTCACGCACACACATATTTCTTCAAAAACC	
FET99‐F3	CAAAGTAACGGCAACAATCA	
FET99‐R3	TTTCGGCACTAAGAACAAAT	
*4fetsΔ/Δ*‐F1	GACCAATGATTGGGTTTAACGATAGTTGGCCGTTACCTATGCCACCTGACGTCTAAGAA	KO *4fetsΔ/Δ copy1* by PCR method
*4fetsΔ/Δ*‐R1	AATGCTTTGTTCTTCCTCCTCCTCATCATCATCATCATC GGCCTTTTGCTCACATGTTC	
*4fetsΔ/Δ*‐F2	CTGGTTGGGTGAATGCTAACCCAGATGGTGGATTTGAAA GACCAATGATTGGGTTTAAC	
*4fetsΔ/Δ*‐R2	TGAAGGGCTAGAAGAGGAACCAGTTGCAGCCTGCTCAGTAATGCTTTGTTCTTCCTCCT	
*4fetsΔ/Δ*‐F3	GTGTTTTGAGTGCAGGTGACGATGCTTCCAATGAATTGGTGCCACCTGACGTCTAAGAA	KO *4fetsΔ/Δ copy2* by PCR method
*4fetsΔ/Δ*‐R3	TCCCTCACCATTGGATATTCTGGCCAATCCGCATGATCGGGCCTTTTGCTCACATGTTC	
*4fetsΔ/Δ*‐F4	ACATTACCTACACGACACCTAGAGTTCCAACTTTGTTGAGTGTTTTGAGTGCAGGTGAC	
*4fetsΔ/Δ*‐R4	CTCATAACAATGTAGGATTGGGGTCTAACGTAAACAGTGTCCCTCACCATTGGATATTC	

### Measurement of melanin production in *C. albicans*

Melanin production was measured according to previously described methods [30, 31] with minor modifications. *C. albicans* cells were cultured overnight in liquid YPD medium at 22 °C. The cell pellets were collected, washed twice and then reinoculated at 2.0 × 10^6^ cells·mL^−1^ into a liquid minimal medium (15.0 mm glucose, 10.0 mm MgSO_4_, 29.4 mm KH_2_PO_4_, 13.0 mm glycine, 3.0 m vitamin B_1_, pH 5.5), with or without 1 mm DOPA, and further incubated at 22 or 37 °C in darkness in a shaker at 200 r.p.m. GlcNAc (5 mm) was added to the minimal medium to stimulate melanin production. Melanin production was approximated by measuring the optical density at 310 nm (OD_310_) or 470 nm (OD_470_) of the whole culture versus a matched blank. Cells grown without DOPA were used as a blank. Melanin production was also approximated by OD_310_ or OD_470_ in resuspended cell pellets. To monitor the visible pigment of melanized cells, we resuspended cells grown with DOPA in PBS and transferred to 96‐well plates for photography.

### Enzyme assay

The oxidative enzyme associated with melanin synthesis in *C. albicans* was determined by a previously reported method [23, 37], and the enzyme solution was prepared as previously described [38, 39]. In brief, overnight‐cultured *C. albicans* cells were reinoculated at 2.0 × 10^6^ cells·mL^−1^ in YPD and cultured at 22 or 37 °C. Then, the cells were washed with cold PBS, and all further procedures were carried out at 4 °C. Cells were resuspended in a lysis buffer (10 mm Tris–HCl, pH 8, 250 mm NaCl, 0.1% Nonidet P‐40, 0.5 mm DTT, 0.5 mm phenylmethylsulfonyl fluoride, 2 mm benzamidine, 0.5 μg·mL^−1^ leupeptin, 1.4 μg·mL^−1^ pepstatin, 2.4 μg·mL^−1^ chymostatin and 17 μg·mL^−1^ aprotinin) and homogenized with acid‐washed glass beads (Sigma, St. Louis, MO, USA) using a Fast‐Prep homogenizer (FP120; Thermo Electron, Waltham, MA, USA) and then centrifuged at 16 200 ***g*** for 15 min. The supernatant was used for the oxidative enzyme activity assay. Protein concentrations of the extracts were measured by the Bradford method, and the melanin‐associated oxidase activity was determined with DOPA as a substrate. Enzyme solutions (10 μL) were incubated in a 200‐μL minimal medium with 1 mm DOPA at 22 or 37 °C for 30 min, and the OD_310_ was recorded. One unit was defined as 0.001 absorbance unit in 30 min of the assay. Enzyme activity was expressed as mU Abs·min^−1^·μg protein^−1^.

### Bone marrow‐derived macrophage induction

Bone marrow‐derived macrophages (BMDMs) were prepared as previously described [40]. In brief, bone marrow cells were obtained by flushing the femur and tibia of Institute of Cancer Research (ICR) mice aged 6–8 weeks with BMDM culture medium (RPMI‐1640 medium containing 10% FBS, 30% L929 conditioned medium and 1% penicillin–streptomycin). After removal of nonadherent cells on day 4, fresh BMDM culture medium was added, and on day 5, BMDMs were seeded into 12‐well plates at 5 × 10^5^ cells per well and used on day 7.

### Phagocytosis of *C. albicans* cells

BMDMs were plated at 5 × 10^5^ cells per well in 12‐well plates and infected with *C. albicans* cells (multiplicity of infection = 3). At 2 h postinfection, after washing away free fungal cells with 1% PBS, *C. albicans* uptake by macrophages was counted by light microscopy after fixation and staining using a modified Wright–Giemsa stain (Sigma) as previously described [41]. Phagocytosis was defined as the percentage of macrophages taking up at least one fungal cell, and the phagocytic index was defined as the number of fungal cells taken up per 100 macrophages. Data are shown as means ± standard deviation (SD) from three independent experiments by analyzing at least 200 macrophages per well. We also used the colony‐forming units (CFUs) method to analyze macrophage phagocytosis [40]. BMDMs were plated at 5 × 10^5^ cells per well in 12‐well plates and infected with *C. albicans* cells (multiplicity of infection = 0.5–3). After 0.5–12 h of coincubation, the wells were washed twice with 1% PBS to remove free fungal cells. BMDMs were scraped, lysed in a lysis buffer (50 mm Tris, pH 7.5, 150 mm NaCl, 1% Triton X‐100, 1 mm EDTA), resuspended, serially diluted and plated onto YPD agar. Fungal CFUs were counted after 48 h of incubation at 30 °C.

### Analysis of melanized *C. albicans* resistance to UV radiation, H_2_O_2_ oxidation and antifungal drugs

*C. albicans* cells were cultured in a minimal medium containing 5 mm GlcNAc with 1 mm DOPA at 22 °C for 4 days and then suspended in 1% PBS. Cells (1.0 × 10^7^) were exposed to UV at 30 000 μJ·cm^−2^ for 30 s or treated with 1.5 mm H_2_O_2_ for 2 h, and then spread onto the YPD plate for survival analysis. Similarly, DOPA‐incubated *C. albicans* cells were suspended in PBS, and 1.0 × 10^7^ cells were treated with 32 μg·mL^−1^ caspofungin or 1 μg·mL^−1^ amphotericin B (AMB) for 2 h. Fungal CFUs were counted after 48‐h incubation at 30 °C.

### Scanning electron microscopy and transmission electron microscopy

Scanning electron microscopy (SEM) and transmission electron microscopy (TEM) were performed according to the methods described by Walker *et al*. [31]. *C. albicans* cells were collected, washed twice with cold PBS, fixed in 2.5% glutaraldehyde in 0.1 m sodium phosphate buffer (pH 7.3) for 24 h at 4 °C, postfixed with 1% OsO_4_ for 1 h and serially dehydrated in ethanol (30%, 50%, 70%, 80%, 95% and 100%) for 10 min. Then, the cells were critical point dried in CO_2_ with a Leica EM CPD300 dryer (FEI Ltd., Hillsboro, USA), sputter‐coated with gold using a Leica EM SCD050 coater (FEI Ltd.) and viewed in a FEI Quanta 250 scanning electron microscope (FEI Ltd.).

*C. albicans* cell pellets were fixed in 2.5% (v/v) glutaraldehyde in 0.1 m sodium phosphate buffer (pH 7.3) for 24 h at 4 °C [31]. After three washes with PBS, the cells were treated with 1% OsO_4_ for 1.5 h, serially dehydrated in ethanol (30%, 50%, 70%, 80%, 95% and 100%) and infiltrated with acetone/Spurr resin. Additional infiltration was provided under vacuum in a mixture of acetone and Epon 812 for 1.5 h. The samples were embedded in Epon 812 and polymerized at 60 °C for 48 h, and further treated with a Leica EM TRIM2 machine (Leica Mikrosysteme GmbH A, Wien, Austria). Semithin survey sections were prepared with a diamond knife on a Leica EM UC7 ultramicrotome and stained with 1% toluidine blue to detect the area with the best cell density. Ultrathin sections (70 nm) were prepared with a diamond knife, stained with uranyl acetate and lead citrate, and observed under a FEI Tecnai G2 Spirit transmission electron microscope (FEI Ltd.).

### Quantitative PCR

RNA extraction, cDNA synthesis and quantitative PCR (qPCR) amplification were performed as described previously [42]. Exponentially growing *C. albicans* cells were collected for RNA extraction. The first‐strand cDNA was synthesized from 3 μg total RNA in a 60‐μL reaction volume using the cDNA synthesis kit (TaKaRa Biotechnology, Dalian, China). qPCRs were performed using the Chromo 4 Real‐Time PCR System (Bio‐Rad, Indianapolis, IN, USA). SYBR Green I (TaKaRa) was used to visualize and monitor the amplified product in real time. Transcription levels of genes in different samples were normalized against the levels of *ACT1*. Primers used are shown in Table 3. The data were measured with three independent experiments.

### Detection of laccase‐like activity in *C. albicans*

Nondenaturing gel electrophoresis was used to detect the laccase‐like activity in *C. albicans* as previously described [30]. *C. albicans* cells were cultured in YPD overnight at 22 °C. After the cells were harvested, the total proteins were extracted with acid‐washed glass beads (Sigma) using a Fast‐Prep homogenizer (FP120; Thermo Electron). The homogenate was centrifuged at 16 200 ***g*** for 30 min. The protein concentration of the cell lysate was measured by the Bradford method, and 300 μg total protein in each 40 μL sample was loaded onto gels. Proteins were also estimated using the Coomassie brilliant blue method (Fig. S2). Commercially, laccase (from *Rhus vernicifera*; Sigma) was used as a positive control, and an equal protein sample was boiled for 5 min as a negative control. *Rhus vernicifera* laccase (40 U equivalent) and 300 μg total protein of *C. albicans* cells were loaded onto gels. After electrophoresis, the gels were immersed in 1 mm l‐DOPA in 0.1 m citric acid/0.2 m Na_2_HPO_4_ (pH 6.0) buffer overnight. Positive laccase activity was revealed by *C. albicans* protein extracts as shown by dark bands, which confirmed that l‐DOPA had polymerized to form melanin.

### Quantitative mating assays in *C. albicans*

Quantitative mating analysis was performed as previously described [43]. YPD or SCD medium was used for mating between the *C*. *albicans MTL* ***a*** and *MTLα* strains. The strains were mixed at 1 : 1 and incubated in YPD plates at 22 °C for 6 days, then spread onto SCD plates without amino acids. The mating efficiency = conjugants/(limiting parent + conjugants) = the greater of (‐Ade‐Ura‐His‐Arg)/(‐Ade) or (‐Ade‐Ura‐His‐Arg)/(‐Ura‐His‐Arg).

## Results

### Opaque cells produce more dark‐pigmented melanin than white cells

**Fig. 1:**
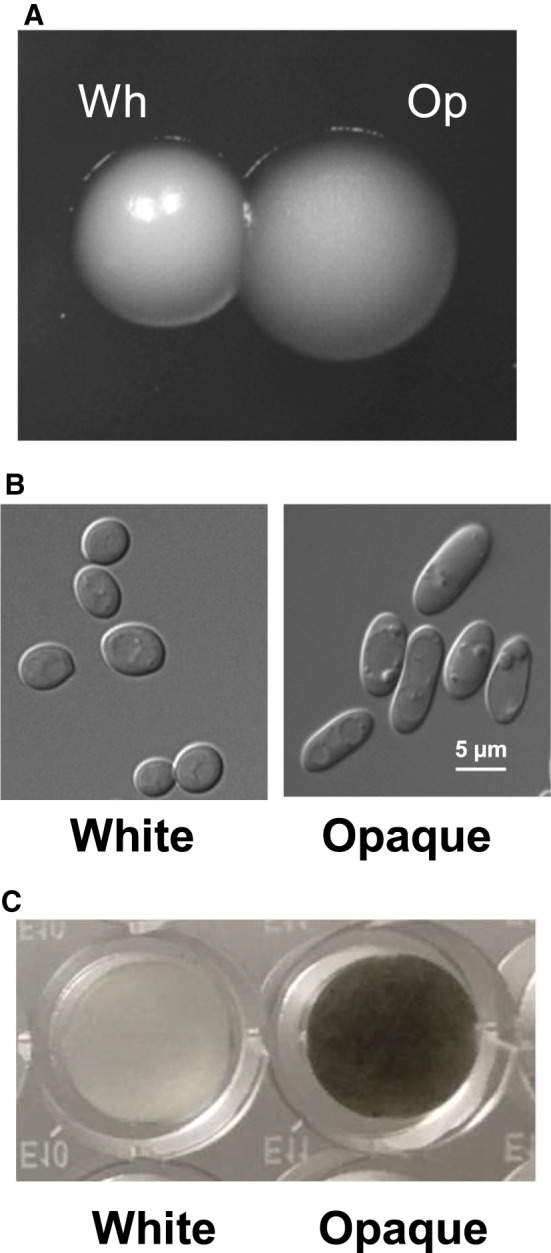
Opaque cells produce more dark‐pigmented melanin. (A) Representative colonies of white and opaque WT *MTL* homozygous strain (JYC5 + pACT1) on YPD solid agar at 22 °C for 5 days. (B) Cell morphology from the white or opaque colonies in (A). Scale bars, 5 μm. (C) Melanin production in white or opaque cells. The white or opaque cells cultured in minimal medium with 1 mm l‐DOPA and 5 mm GlcNAc at 22 °C for 4 days, then pelleted, resuspended with PBS in 96‐well plates and photographed. Op, opaque; Wh, white.

**Fig. 2:**
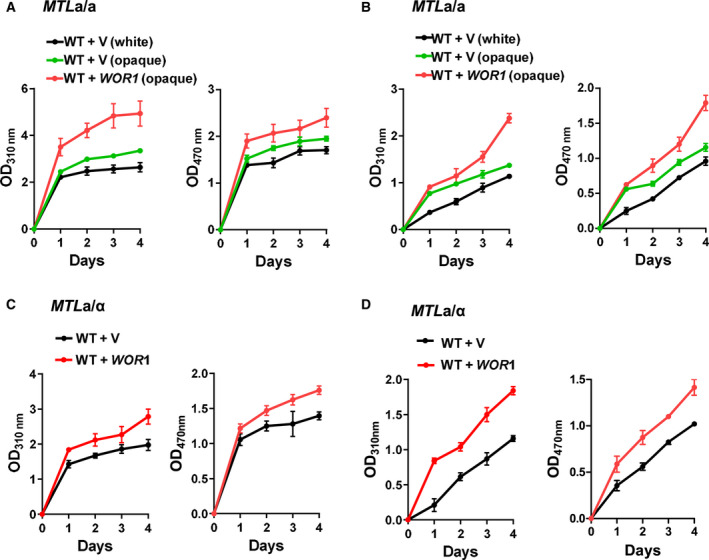
Melanin production in *MTL* homozygous and heterozygous cells. Melanin production by *MTL* ***a/a*** WT (JYC5 + pACT1) white or opaque cells, and *WOR1* overexpression (JYC5 + pACT1‐WOR1) opaque cells were determined by OD_310_ or OD_470_ for whole cultures (A) and in resuspended cell pellets (B). Melanin production by *MTL* ***a*** */α* WT (CAI4 + pACT1) or *WOR1* overexpression (CAI4 + pACT1‐WOR1) cells for whole cultures (C) and in resuspended cell pellets (D). The overnight cultures of *C. albicans* cells were collected, washed and grown in minimal medium supplemented with 1 mm DOPA and 5 mm GlcNAc at 22 °C in darkness. The samples were taken at daily intervals, and OD_310_ or OD_470_ was measured against matched cell suspensions grown without DOPA for melanin estimation. Data represent the mean ± SD (*n* ≥ 3 independent experiments).

*C. albicans* was featured as a white yeast and was later found to exist in an opaque form [4]. *C. albicans* can switch between white and opaque cellular phenotypes to survive at different host niches. The white colony appears white and hemispherical, whereas the opaque colony appears dark and flat (Fig. 1A). The white cells appear round, whereas the opaque cells are elongated (Fig. 1B). To investigate whether the dark color of the opaque colony was correlated with melanin pigment production in *C. albicans*, we measured melanin pigment in white or opaque cells with l‐DOPA as a substrate using previously reported methods [30, 31] and found that opaque cells produced more melanin pigment than white cells. After 4‐day incubation at 22 °C in a defined liquid minimal medium containing 1 mm DOPA and 5 mm GlcNAc, the resuspended opaque cell pellets showed a black color, while the white cell pellets showed a white color (Fig. 1C), suggesting that opaque cells produced more melanin pigment than white cells. To quantify the melanin production, we measured the OD of opaque cells in whole cultures (Fig. 2A) and cell pellets (Fig. 2B) at OD_310 nm_ and OD_470 nm_; opaque cells grown with DOPA showed a higher absorption value than white cells.

### Wor1 promotes melanin production in both *MTL* homozygous and heterozygous cells

Wor1 is a master regulator for white‐to‐opaque switching in *C. albicans* [12, 13, 14], and this white‐to‐opaque switching is coupled with the sexual mating process under ***a***1–*α*2 repression [44]. The ability to switch to the opaque state depends on whether the cells are homozygous for the *MTL* that controls the cell type [43]. In *MTL* ***a/a*** or *MTLα/α* homozygous cells, ectopically expressed Wor1 induces the transcription of endogenous *WOR1* in a positive feedback manner, promotes white‐to‐opaque switching and locks cells in the opaque phase [12]. In *MTL* ***a*** */α* heterozygous cells, overexpression of Wor1 had lower efficiency in white‐to‐opaque switching [12]. We first tested the impact of Wor1 on melanin production in *MTL* ***a/a*** homozygous cells. Compared with white cells, the wild‐type (WT) opaque cells produced more melanin in the DOPA‐containing medium. At both OD_310 nm_ (Fig. 2A,B, left) and OD_470 nm_ (Fig. 2A,B, right), the opaque cells grown with DOPA showed a higher absorption value than the white cells during the 4‐day incubation. The opaque cells produced more melanin than the white cells in whole cultures (Fig. 2A) or cell pellets (Fig. 2B). Interestingly, when we used a *WOR1* overexpression strain (WT + *WOR1*) in which the endogenous *WOR1* was positively induced by ectopic Wor1 and the opaque phase was stabilized, the *WOR1‐*stabilized opaque cells showed even higher absorption values than the WT opaque cells (WT + vector; Fig. 2A,B). This phenomenon is probably due to the unstable opaque state of the WT opaque cells in the minimal medium used for melanin production. Therefore, we used *WOR1* overexpression‐stabilized opaque cells (WT + *WOR1*) for later analyses. We tested the impact of Wor1 on melanin production in *MTL* ***a*** */α* heterozygous cells. Because it is not possible to maintain the WT *MTL* ***a*** */α* heterozygous cells in an opaque state in air conditions *in vitro*, we used ectopic Wor1 formed *MTL* ***a*** */α* opaque cells to measure the melanin production. As expected, *WOR1* overexpression‐stabilized *MTL* ***a*** */α* opaque cells (WT + *WOR1*) produced more melanin than the WT *MTL* ***a*** */α* white cells (WT + vector) when they were incubated in the DOPA‐containing medium and showed a higher absorption value at OD_310 nm_ or OD_470 nm_ in whole cultures (Fig. 2C) and cell pellets (Fig. 2D), indicating that Wor1 promoted melanin production in both *MTL* homozygous and heterozygous cells. However, *WOR1* overexpression promoted less melanin production in *MTL* ***a*** */α* heterozygous cells than in *MTL* homozygous cells, reflecting the impact of ***a***1–*α*2 repression on melanin production.

### The pimpled surface of opaque cells is correlated with externalization of melanin

**Fig. 3:**
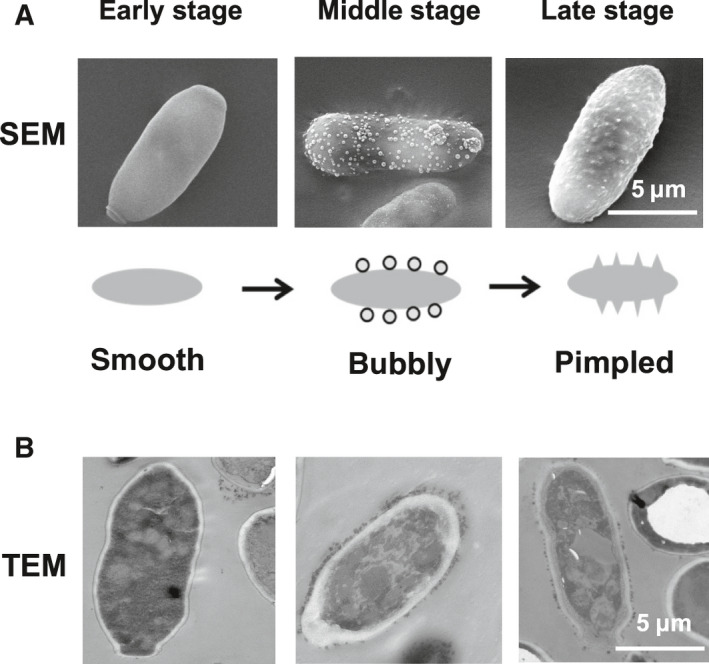
Examination of opaque cell surfaces. (A) Scanning electron micrographs of WT *MTL* homozygous strain overexpressed with *WOR1* (JYC5 + pACT1‐WOR1). Opaque cells were cultured in minimal medium with 1 mm DOPA and 5 mm GlcNAc at 22 °C and sampled at early stage (~ 6 h), middle stage (~ 4 days) or late stage (~ 10 days). Schematic diagram of cell surface correlated with melanin externalization during the cell growth is shown beneath the micrographs. (B) Transmission electron micrographs of opaque cells cultured in DOPA‐containing media and sampled at the three stages. Scale bars, 5 μm.

White and opaque cells have distinct features [4]. Opaque cells are elongated with pimples on the surface, forming flat and opaque colonies. The mechanisms that control the dark color of opaque colonies and the pimpled surface of opaque cells are unknown. We speculated that the dark color and pimpled surface may be associated with melanin synthesis and externalization. To analyze the secretion of melanin, we collected homozygous *WOR1*‐overexpressing opaque cells (*MTL* ***a/a*** WT + *WOR1*) and incubated them with DOPA for a 10‐day growth period to observe melanin externalization by SEM and TEM using the method described previously [31, 45]. SEM showed several types of opaque cell surfaces during the process of melanin externalization. Opaque cells exhibited a smooth surface during the early growth stage in the log phase (Fig. 3A, left), a bubbly form during the middle growth stage after 4‐day incubation (Fig. 3A, middle) and a pimpled surface during the late growth stage after 10‐day incubation (Fig. 3A, right). TEM also showed different patterns of melanin externalization corresponding to the three opaque growth stages. Most of the dark flecks remained inside the opaque cells during the early stage (Fig. 3B, left); dark melanin particles were secreted constitutively outside the opaque cells during the middle stage (Fig. 3B, middle), and fewer dark particles were observed both inside and outside the opaque cells in the late stage (Fig. 3B, right). Walker *et al*. [31] reported that the presence of exogenous substrate DOPA is required for the externalization of melanin in *C. albicans*. Melanin particles were not observed in DOPA‐free cultures [31]. To verify that the small dark flecks from opaque cells are indeed melanin, we compared the TEM images of opaque cells grown with or without the addition of DOPA for 4 days (Fig. S3). As expected, the cells cultured with DOPA were surrounded by melanin clumps, whereas melanin clumps were not observed in cells cultured in DOPA‐free media. All these SEM and TEM findings suggest that the cell surface appearance was associated with melanin synthesis and externalization. During the early growth period, melanin was synthesized but barely secreted, and the opaque cell surface appeared smooth. With the increase in melanin secretion during the middle stage, the opaque cells presented a bubbly form. With the exhaustion of melanin externalization during the late stage, opaque cells exhibited a pimpled surface. Therefore, the pimpled surface of opaque cells described previously could be correlated with complete melanin externalization.

### Fets function as pigment MCOs for melanin production in *C. albicans*

**Fig. 4:**
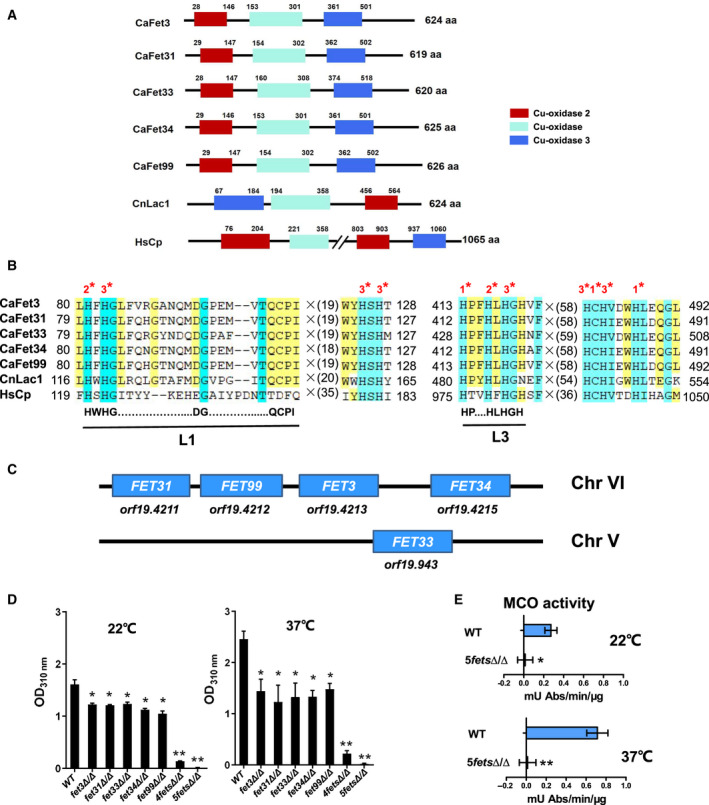
Fets are required for melanin production in *C. albicans*. (A) Schematic diagram of putative MCOs from *C. albicans* and other Cu‐oxidase domain‐containing proteins in *C. neoformans* and *H. sapiens*. Sequences of *C. albicans* MCOs (CaFet3, CaFet31, CaFet33, CaFet34 and CaFet99), *C. neoformans* laccase (CnLac1) and HsCp were obtained from the NCBI database (https://www.ncbi.nlm.nih.gov/). (B) Amino acid sequence comparison of four conserved regions of the Cu‐oxidase domain‐containing proteins. Red asterisks indicate residues that are conserved in the copper ion binding domain. Potential coordination sites for the three different types of copper ions are indicated by 1*, 2* and 3*. (C) Four *FET* genes are clustered on chromosome 6 in the tandem order of *FET31‐FET99‐FET3‐FET34*, and one single gene, *FET33*, is located on chromosome 5. (D) Melanin production in the *MTL* homozygous *fets* deletion mutant strains. *C. albicans* white cells of WT (JYC5) (***a/a***), *fet3Δ/Δ* (CDB2) (*ɑ/ɑ*), *fet31Δ/Δ* (CDB4) (***a/a***), *fet33Δ/Δ* (CDB5) (***a/a***), *fet34Δ/Δ* (CDB6) (***a/a***), *fet99Δ/Δ* (CDB7) (***a/a***), *4fetsΔ/Δ* (CDB9) (***a/a***) and *5fetsΔ/Δ* (CDB11) (***a/a***) were cultured in minimal medium with 1 mm DOPA and 5 mm GlcNAc at 22 or 37 °C for 4 days, and the whole cultures were measured at 310 nm. The *fet33Δ/Δ* and *fet34Δ/Δ* mutants were introduced with a vector pPR671, and the other strains were transformed with a vector pBA1. The melanin production of each strain was detected by OD_310 nm_ and corrected the value by its own negative control cultured without DOPA. (E) Melanin‐associated oxidase activity in WT (JYC5 + pBA1) and *5fetsΔ/Δ* mutant (CDB11 + pBA1) cells. The white cells were cultured in YPD at 22 or 37 °C and collected for extraction of total proteins. Specific oxidase activity was determined using DOPA as a substrate and indicated as mU Abs·min^−1^·μg protein^−1^. Bars, mean ± SD. Data are representative of at least three independent experiments, each with similar results. Data relative to OD_310_ in WT cells. *P < 0.05, **P < 0.01, by Student's *t*‐test. aa, amino acids.

Knowing that melanin production is usually catalyzed by a polyphenol oxidase (laccase) with an exogenous diphenolic as a substrate [30, 31], we searched the *C. albicans* genome database (http://www.candidagenome.org) and found five predicted MCOs, which have been previously reported as Fets annotated as Fet3, Fet31, Fet33, Fet34 and Fet99 [29]. All five Fets contain three multicopper binding domains that are conserved in Fets such as *Saccharomyces cerevisiae* Fet3 [46] and laccases such as *C. neoformans* Lac1 (CnLac1) [23]. The conserved multicopper domains also exist in *Homo sapiens* ceruloplasmin (HsCp; Fig. 4A). As expected, the conserved histidine and cystatin residues required for copper binding were found in the multicopper domains, and all potential coordination sites for the three different types of copper ions were found in these regions (Fig. 4B). Interestingly, laccase‐specific signature sequences, namely, L1 and L3, were present in the five Fets and CnLac1 (Fig. 4B). However, the L1 pattern was absent in HsCp. The proposed patterns L1 (H‐W‐H‐G‐x(9)–D‐G‐x(5)–Q‐C‐P‐I) and L3 (H‐P‐x‐H‐L‐H‐G‐H) were suggested to be specific for laccases and proposed to distinguish laccases from other MCOs [26].

To determine whether the five Fets could function as laccase‐like CnLac1, we constructed a series of *MTL* homozygous Fet deletion mutants by PCR‐based homologous recombination [32]. Because four of the five Fet‐encoding genes were clustered in a tandem order of *FET31‐FET99‐FET3‐FET34* (*orf19.4211*, *orf19.4212*, *orf19.4213* and *orf19.4215*) on chromosome 6, and *FET33* (*orf19.943*) was located on chromosome 5 alone (Fig. 4C), we constructed five single *FET* gene deletion mutant strains, including *fet3Δ/Δ, fet31Δ/Δ, fet33Δ/Δ, fet34Δ/Δ* and *fet99Δ/Δ*, four *FET* genes deleting mutant strain *4fetsΔ/Δ* (*FET31‐FET99‐FET3‐FET34*) and five *FET* genes deleting mutant strain *5fetsΔ/Δ* for functional analysis and examined the ability of these mutant strains in melanin production using DOPA as a substrate. Knowing that white cells can also produce melanin, we cultured white phase cells in minimal medium with 1 mm DOPA and 5 mm GlcNAc and used the whole culture for measurement to simplify the procedures. Then, we measured the OD_310_ and found that pigment production was reduced in each *fet* single gene with the mutant depleted, especially when the strains were cultured at 37 °C (Fig. 4D), which reflects the contribution of each Fet to pigment oxidase activities. When the four clustered *FET* genes were deleted (*4fetsΔ/Δ*), pigment production was significantly decreased. The OD_310_ was reduced 10‐fold in the *4fetsΔ/Δ* mutant compared with the WT when the cells were cultured at 22 °C and 13‐fold when the cells were cultured at 37 °C (Fig. 4D). After depletion of all five *FET* genes (*5fetsΔ/Δ*), pigment production was completely blocked (Fig. 4D). These results suggest that each Fet contributed to the activity of the pigment MCO. We further measured the global melanin‐associated oxidase activity of these mutant cells using DOPA as a substrate. Specific oxidase activity was indicated as mU Abs·min^−1^·μg protein^−1^ [23, 37]. We found that melanin‐associated oxidase activity was completely abolished in the *5fetsΔ/Δ* mutant when the cells were cultured at 22 or 37 °C (Fig. 4E). These data suggest that all five Fets function as pigment MCOs associated with melanin synthesis in *C. albicans* using exogenous phenolic substrates, and the four clustered *FET* gene products play major roles in melanin pigment production.

In this study, we used CAI4 background strain for Fet‐mediated melanin analysis, because a gene, *IRO1*, which is involved in iron metabolism, is mutated in CAI4, although the CAI4 strain is derived from a WT strain SC5314 [36]. To avoid misunderstanding the functional role of Fets in melanin production, we compared the melanin production in SC5314 and CAI4 background strains. We screened and selected the *MTL* ***a***/***a*** homozygous strain of SC5314, the parent of CAI4, containing a WT *IRO1* gene. In white cells, the melanin levels produced in SC5314 *MTL* ***a***/***a*** strain are similar to that in the CAI4 *MTL* ***a***/***a*** strain (Fig. S4A,B). In opaque cells, the melanin productions are also similar in the two *MTL* ***a***/***a*** homozygous strains (Fig. S4C,D). We further measured the melanin productions in *MTL* ***a*** */α* heterozygous strains of SC5314 and CAI4; the melanin productions are almost the same in these two heterozygous strains (Fig. S4E,F). Furthermore, we examined the MCO activities in the two *MTL* ***a***/***a*** homozygous strains. The MCO activities from the SC5314 *MTL* ***a***/***a*** strain are almost equal to that of the CAI4 *MTL* ***a***/***a*** strain at both 22 and 37 °C (Fig. S5). Thus, absence of the *IRO1* gene seems to have no obvious effect on the melanin production in the conditions we used. Considering the technical difficulty, and consistent with our previous results, we choose CAI4 or BWP17 and their homozygous descendants for the melanin production analysis.

### Wor1 up‐regulates *FET* expression in rich medium at physiological temperature of 37 °C

**Fig. 5:**
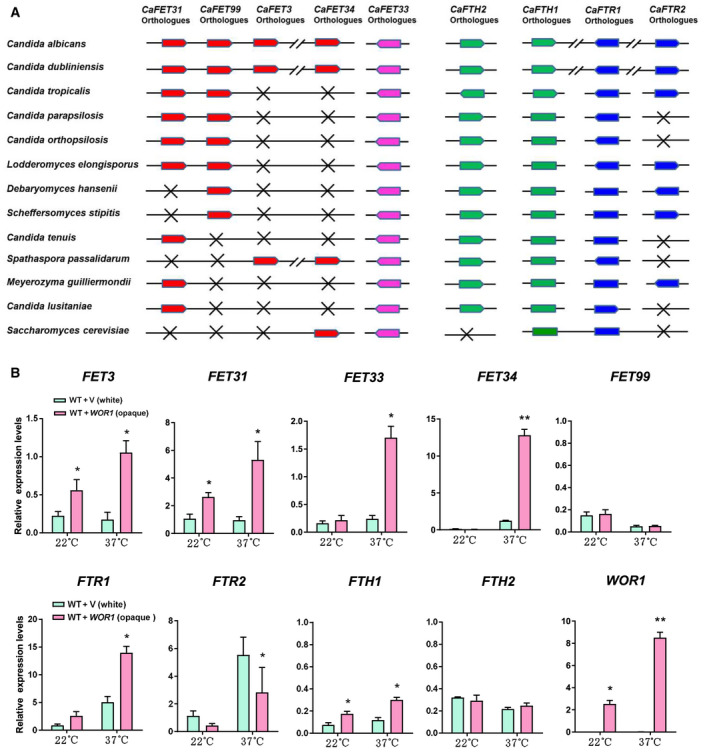
Wor1 up‐regulates *FET* expression in YPD media. (A) Predicted Fet orthologs in *Candida* species (http://www.candidagenome.org). (B) Expression of *FETs*, *FTRs* and *FTHs* in WT (JYC5 + vector) white and WT with *WOR1* (JYC5 + *WOR1*) opaque cells. The white or opaque cells were cultured in YPD at 22 °C overnight, then spread onto YPD plates and incubated at 22 or 37 °C for 1 day. The cells were collected for RNA extraction and further for qRT‐PCR analysis. The signal obtained from *ACT1* mRNA was used as a loading control for normalization. Bars, mean ± SD. Data are representative of at least three independent experiments, each with similar results. Data relative to gene expression level in WT cells at the same temperature. *P < 0.05, **P < 0.01, by Student's *t*‐test. V, vector.

Fet homologs were found in all *Candida* species. However, the four‐gene *FET* clusters were present in only two *Candida* species, *C. albicans* and *C. dubliniensis*, and were absent in other *Candida* species or closely related fungal species, such as *S*. *cerevisiae* (Fig. 5A). The existence of the four‐gene Fet clusters in *C. albicans* and *C. dubliniensis* was probably correlated with melanin pigment production. *FET33* homologs, however, existed in all *Candida* species and *S. cerevisiae*. Conversely, the four permeases encoding genes *FTR1*, *FTR2*, *FTH1* and *FTH2* were broadly present in *Candida* species, unlike that in *S. cerevisiae*, which had only two iron permeases encoding genes, *FTR1* and *FTH1*. Therefore, the existence of Fet clusters and multiple permease homologs in opportunistic pathogenic fungi is of evolutionary importance.

Knowing that Wor1 promoted melanin production in opaque cells (Fig. 2), we then tested the impact of the master regulator Wor1 on the expression of MCO encoding genes. Interestingly, all five *FET* promoters contained predicted Wor1 binding sites (Fig. S6). To determine whether Wor1 regulated *FET* gene transcription, we used *MTL* homozygous *WOR1‐*stabilized opaque cells (WT + *WOR1*) that highly expressed *WOR1* and WT white cells (WT + vector) that did not express *WOR1* (Fig. 5B), to detect the mRNA levels of all five *FET* genes in *C. albicans* cells cultured in a YPD‐rich medium. We found that *WOR1* overexpression up‐regulated *FET3* and *FET31* transcription at 22 °C but had a subtle effect on the other *FET* genes (Fig. 5B). At the human physiological temperature (37 °C), *WOR1* overexpression up‐regulated the expression of the *FETs* more obviously and increased the transcription of *FET3, FET31*, *FET33* and *FET34* by 6‐, 5.7‐, 7.5‐ and 11‐fold, respectively (Fig. 5B, upper panels). Except for *FET99*, all four other *FET* genes were up‐regulated by Wor1 at 37 °C. The promoters of four permease‐encoding genes, *FTR1*, *FTR2*, *FTH1* and *FTH2* [29], also contained the predicted Wor1 binding sites (Fig. S6). We subsequently examined the transcription level of the permease‐encoding genes in *WOR1‐*stabilized opaque cells and WT white cells. It was found that *WOR1* overexpression up‐regulated *FTR1* and *FTH1* transcription at 37 °C and down‐regulated *FTR2* transcription but had a subtle effect on *FTH2* transcription (Fig. 5B, lower panels). Our data suggest that Wor1 enhances the expression of *FET* genes in rich medium, especially at the human physiological temperature of 37 °C.

### Wor1 up‐regulates *FET* expression in GMM‐LA

**Fig. 6:**
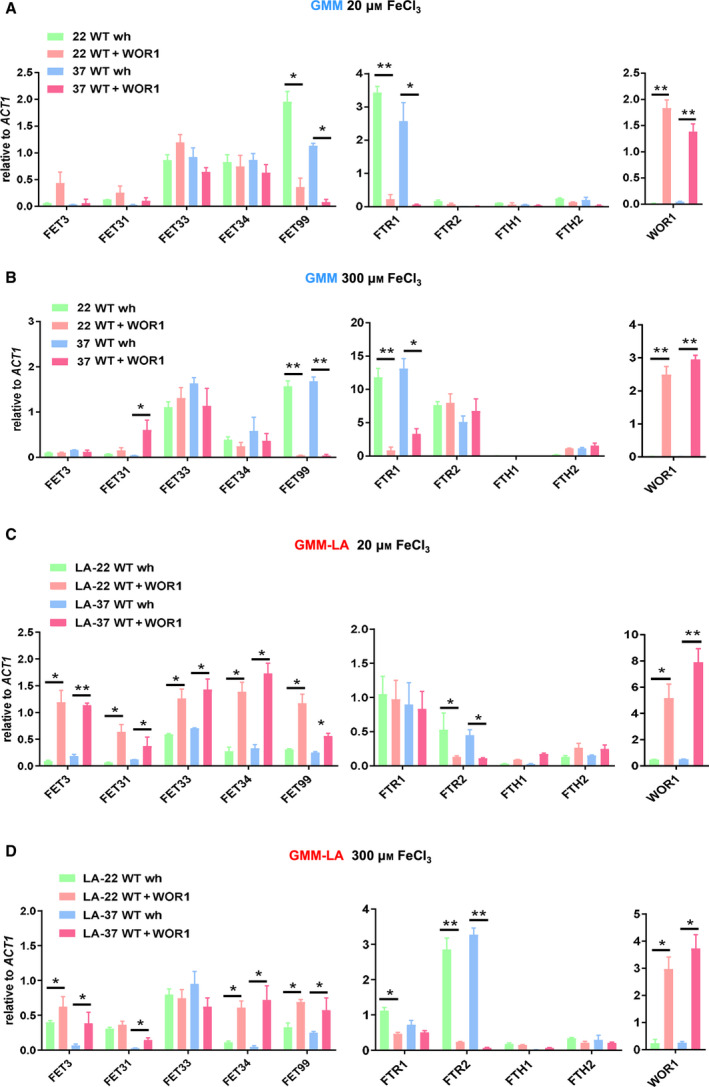
Distinct expression patterns of *FET* genes in glucose minimal media (GMM). WT (JYC5 + vector) white cells and *WOR1* overexpression (JYC5 + *WOR1*) opaque cells were cultured in YPD overnight at 22 °C and then released into GMM [38 mm (NH4)_2_SO_4_] (A, B) or with LA (GMM‐LA) [25 μm (NH4)_2_SO_4_] (C, D) for 6 h at 22 °C or 3 h at 37 °C. The cells were collected and extracted for qRT‐PCR analysis. To prepare the media containing different concentrations of iron, we first depleted GMM or GMM‐LA of iron by adding 1 mm ferrozine and 10 mm ascorbic acid, followed by adding different amounts of FeCl_3_. Bars, mean ± SD. Data are representative of at least three independent experiments, each with similar results. Data are relative to gene expression level in WT cells at the same temperature. *P < 0.05, **P < 0.01, by Student's *t*‐test.

Previous reports showed that the five Fets had distinct iron‐dependent regulation patterns in response to iron concentration [29]. Unlike the growth condition of opaque cells, we used GMM to test the effect of iron on *FET* expression and analyzed the impact of Wor1 on *FET* expression in the minimal medium with a low (20 μm FeCl_3_) or high (300 μm FeCl_3_) iron concentration. Overnight‐cultured WT white cells or *WOR1‐*stabilized opaque cells from YPD medium were released into the iron‐chelated minimal medium supplemented with FeCl_3_ and grown at 22 °C for 6 h or at 37 °C for 3 h. It was found that the *FET* expression pattern in the GMM was different from that in YPD‐rich medium (Fig. 6). In the GMM with regular ammonium sulfate (38 mm), Wor1 down‐regulated *FET99* expression in an iron‐independent manner but had a subtle effect on the expression of other *FETs* (Fig. 6A,B). We further examined the impact of Wor1 on *FET* expression in the minimal medium with LA sulfate (25 μm GMM‐LA). Interestingly, all five *FET* genes were up‐regulated by Wor1 in GMM‐LA with 20 μm FeCl_3_, and this up‐regulation was independent of growth temperature (Fig. 6C). In GMM‐LA with 300 μm FeCl_3_, three *FET*s (*FET3*, *FET34* and *FET99*) were found to be up‐regulated by Wor1 (Fig. 6D), indicating that *FET* genes were up‐regulated by Wor1 in minimal medium with LA sulfate. Among the *FETs*, the *FET33* expression level was relatively high in the GMM with high ammonium or GMM‐LA, independent of iron concentrations. Expression of the other *FETs* varied depending on the growth conditions. Among the permease‐encoding genes, Wor1 had a repressive effect on *FTR1* in high‐ammonium medium (Fig. 6A,B), but a repressive effect on *FTR2* in LA medium (Fig. 6C,D). Based on the earlier‐mentioned data, we concluded that the expressions of the *FET* and permease‐encoding genes were controlled in multiple layers in response to different environmental stimuli.

### CO_2_ and GlcNAc induce *FET* expression and melanin production

**Fig. 7:**
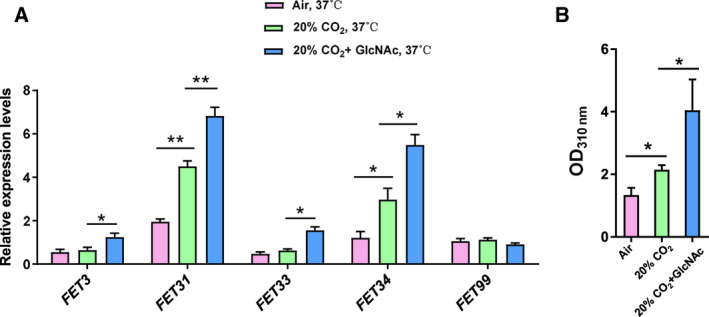
*FET* expression and melanin production in CO_2_ and GlcNAc conditions. (A) The WT (JYC5 + vector) white cells were spread onto YPD or YPG (2% GlcNAc) plates and incubated at 37 °C for 1 day in air or 20% CO_2_. The cells were collected for qRT‐PCR analysis. The expression levels of *FETs* were normalized by *ACT1*. (B) The WT (JYC5 + vector) white cells were grown in liquid minimal media in 10‐cm dishes at 37 °C for 4 days in air or 20% CO_2_ with or without 5 mm GlcNAc. Melanin production was measured at OD_310_. Bars, mean ± SD. Data are representative of at least three independent experiments each with similar results. *P < 0.05, **P < 0.01, by Student's *t*‐test.

Knowing that the physiological level of CO_2_ induced white‐opaque switching and stabilized the opaque phenotype at 37 °C [47], we analyzed the impact of CO_2_ on the expression of MCO encoding genes and melanin production and found that the transcription levels of *FET31* and *FET34* increased significantly when the *MTL* homozygous WT white cells were exposed to a high CO_2_ concentration (20% CO_2_) on YPD plates at 37 °C for 1 day, when compared with the air condition (0.03% CO_2_). However, the *FET3*, *FET33* and *FET99* expression levels remained unchanged significantly (Fig. 7A). Consistently, higher melanin production was detected at a high CO_2_ concentration (Fig. 7B).

GlcNAc was reported to be another white‐opaque switching inducer [48] that promoted *C. albicans* melanin production [31]. The combination of GlcNAc and CO_2_ even induced white‐opaque switching in *MTL* ***a*** */α* heterozygous cells [7]. Thus, we tested the effect of GlcNAc on *FET* gene expression. After 1 day of treatment with GlcNAc on YPG plates in 20% CO_2_, the *FET31* and *FET34* mRNA levels were dramatically increased to a level higher than that with 20% CO_2_ alone. *FET3* and *FET33* expression were also increased; however, *FET99* remained unchanged significantly (Fig. 7A). More melanin production was detected under high CO_2_ condition and GlcNAc (Fig. 7B). Our data showed that a high CO_2_ concentration together with GlcNAc promoted melanin production by increasing the expression level of the *FET* genes at the host physiological temperature of 37 °C.

### Fets contribute to the dark color, but not the elongated shape of opaque cells

**Fig. 8:**
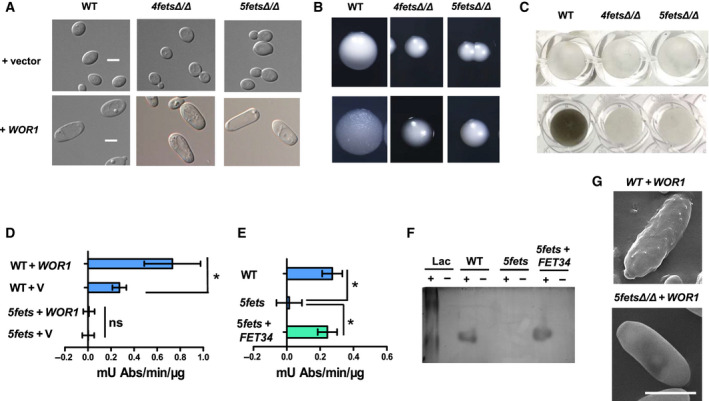
Fets contribute to the dark color, but not the elongated shape of the opaque cells. (A) Cell morphology of the *WOR1* overexpression in WT, *4fetsΔ/Δ* and *5fetsΔ/Δ* mutant strains. The opaque forms of WT (JYC5), *4fetsΔ/Δ* (CDB9) and *5fetsΔ/Δ* (CDB11) with *WOR1* overexpression vector (pACT1‐WOR1) were incubated on a YPD plate in air for 5 days at 22 °C. The white forms of the strains with an empty vector (pACT1) were used as controls. Scale bars, 5 μm. (B) Colony morphology of the strains used in (A). (C) Melanin production of the *WOR1* overexpression in WT, *4fetsΔ/Δ* and *5fetsΔ/Δ* mutant strains. The strains used in (A) were cultured in minimal media with 1 mm DOPA and 5 mm GlcNAc at 22 °C for 4 days, then pelleted and resuspended with PBS in 96‐well plates for photograph. (D) Global melanin‐associated oxidase activities of the *WOR1* overexpression in WT and *5fetsΔ/Δ* mutant strains. The white cells (strains carrying a vector) and *WOR1*‐stabilized opaque cells (strains carrying an ectopically expressed *WOR1*) were used for analysis. The cells were cultured in YPD at 22 °C and collected for extraction of total proteins. Specific oxidase activity was determined with DOPA as a substrate and indicated as mU Abs·min^−1^·μg protein^−1^. (E) Complementation of *FET34* in *5fetsΔ/Δ* mutant. The white cells of WT, *5fetsΔ/Δ* mutant and *5fetsΔ/Δ* with pBA1‐FET34 were cultured for analysis of global melanin‐associated oxidase activity. (F) Nonreducing SDS‐polyacrylamide gel‐containing protein extracts of *Candida albicans* developed with l‐DOPA. The white cells of WT, *5fetsΔ/Δ* mutant and *5fetsΔ/Δ* with pBA1‐FET34 were cultured in YPD and collected for extraction of total proteins. Protein (300 μg) was loaded onto gels and immersed in 1 mm l‐DOPA. Each of the samples was boiled for 5 min to inactivate the enzyme activity. A commercial laccase (40 U equivalent) was loaded as a positive control. +, untreated sample; −, boiled sample. (G) Scanning electron micrographs of *WOR1* overexpression opaque cells in WT and *5fetsΔ/Δ* mutant. Opaque cells were cultured in minimal medium with 1 mm DOPA and 5 mm GlcNAc at 22 °C and sampled at late stage (~10 days). Bars, mean ± SD. Data are representative of at least two (D, E) independent experiments each with similar results. *P < 0.05, by Student's *t*‐test. ns, no significance.

*C. albicans* opaque cells exhibit an elongated shape, and opaque colonies exhibit a dark color [4]. We found that the dark color of the opaque colonies was associated with increased melanin pigment formation and Wor1‐induced up‐regulation of *FET* expression. We then investigated whether the elongated form of the opaque cells was also correlated with Fet expression. To promote and maintain the ‘opaque’ phase, we introduced ectopically expressed Wor1 into homozygous WT cells and *4fetsΔ/Δ* and *5fetsΔ/Δ* mutant cells to induce their endogenous *WOR1*. Similar to WT cells, Wor1‐induced *4fetsΔ/Δ* or *5fetsΔ/Δ* mutant cells exhibited an elongated shape, which is different from the non‐Wor1‐induced round‐shaped white form (Fig. 8A). We also observed colonial color. Unlike the dark and flat WT opaque colonies, *WOR1*‐promoted *4fetsΔ/Δ* and *5fetsΔ/Δ* mutants exhibited smooth, white and small hemispherical colonies (Fig. 8B). The colonies of the *WOR1*‐overexpressing *4fetsΔ/Δ* and *5fetsΔ/Δ* retained the white‐looking shape, but smaller. Thus, Wor1‐induced up‐regulation of the Fets contributed to the dark color, but not to the elongated shape of opaque cells.

To confirm that the dark color formation was due to Fet‐mediated melanin pigment production, we cultured the *WOR1*‐overexpressed WT cells and *4fetsΔ/Δ* and *5fetsΔ/Δ* mutant cells in liquid minimal medium at 22 °C and detected pigment production using DOPA as the substrate. Compared with the WT cell pellets that turned black, the cell pellets of *4fetsΔ/Δ* and *5fetsΔ/Δ* mutants overexpressing *WOR1* remained white (Fig. 8C), resembling the non‐Wor1‐induced WT white cells. We further measured the global melanin‐associated oxidase activities using DOPA as the substrate. In minimal medium with 1 mm DOPA and 5 mm GlcNAc at 22 °C, *WOR1* overexpression WT opaque cells induced a higher melanin‐associated oxidase activity, reaching 0.732 mU Abs·min^−1^·μg protein^−1^, which was approximately 2.7‐fold higher than that from the non‐Wor1‐induced WT white cells (Fig. 8D). In the *5fetsΔ/Δ* mutant, the melanin‐associated oxidase activity was completely depleted, and *WOR1* overexpression could not restore the enzyme activity (Fig. 8D). The depleted melanin‐associated oxidase activity of the *5fetsΔ/Δ* mutant could be restored by reintroducing *FET34* back to the mutant (Fig. 8E). To determine the function of Fets as melanin‐associated oxidases, we performed *in vitro* biochemical assays. The total protein extracts of *C. albicans* opaque phase cells were loaded into nonreducing SDS‐polyacrylamide gels and incubated with l‐DOPA. In comparison with a commercial laccase, positive laccase activity was detected by the protein extracts of WT cells, as shown by a dark band (Fig. 8F), demonstrating that l‐DOPA polymerized to form melanin. The extracts from the *5fetsΔ/Δ* mutant cells did not show a dark band, and the extracts from the *FET34* complementary strain showed a dark band (Fig. 8F), demonstrating the laccase activity of the reintroduced Fet34. Among the four plasma membrane (PM) Fets, Fet34 is the main PM Fet for iron acquisition under iron‐limiting conditions [29]. Considering the high similarities but distinct expression patterns among the four PM Fets, we chose Fet34 under the control of a constitutive promoter (*ADH1p*) for complementation assays. Consistent with its Fet activity, Fet34 showed melanin‐associated oxidase activity (Fig. 8E,F). Using SEM, we further compared the cell surfaces of WT opaque and *5fetsΔ/Δ* mutant ‘opaque’ cells from the late growth stage. As expected, the WT opaque cells showed a pimpled surface, whereas the *5fetsΔ/Δ* mutant ‘opaque’ cells showed a smooth surface (Fig. 8G), indicating that the pimpled surface of opaque cells was associated with melanin synthesis and externalization. We also tested the effects of Fets on mating efficiency. The quantitative mating assay was performed as described previously [43]; *MTL* ***a*** *5fets* mutant cells showed no significant difference with *MTL* ***a*** WT when mating with *MTLα* WT white or opaque cells (Fig. S7), reflecting no impact of Fets on mating processes. These data suggest that the effects of Wor1 on the opaque cell shape and opaque colony color were regulated by two different mechanisms. Wor1‐up‐regulated Fets contributed to the dark color, but not to the elongated shape of the opaque cells.

### Melanization protects *C. albicans* against BMDM phagocytosis and oxidative killing

**Fig. 9:**
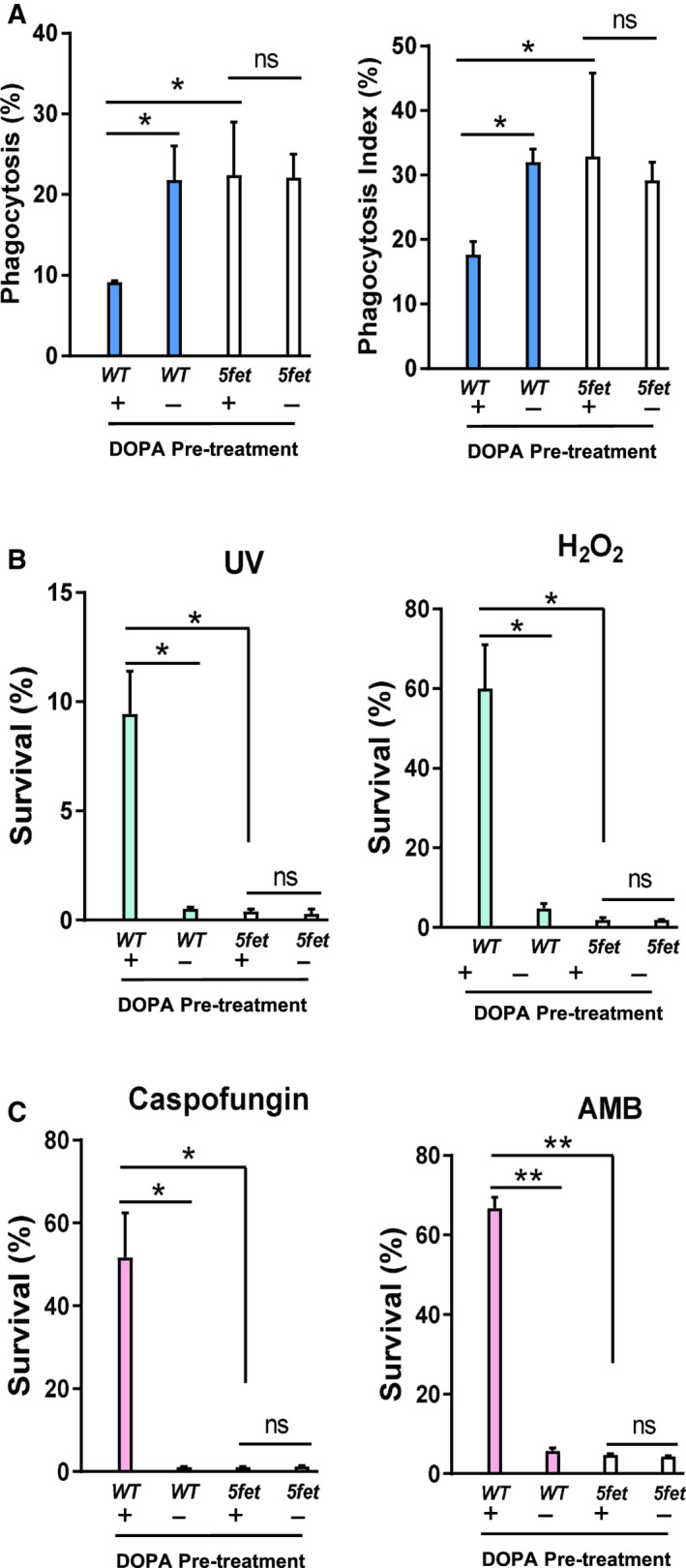
Resistance analysis of melanized *C. albicans* to BMDM phagocytosis, UV radiation, H_2_O_2_ oxidation and antifungal drugs. (A) Melanized *C. albicans* are more resistant to BMDM phagocytosis. The WT (JYC5 + V) and *5fetsΔ/Δ* (CDB11 + V) white cells were cultured in minimal medium containing 5 mm GlcNAc with or without 1 mm DOPA at 22 °C for 4 days, then collected and infected with BMDMs in a 3 : 1 ratio (*C. albicans*: macrophages). After 2‐h coincubation, the phagocytosis (percentage of macrophages that have taken up at least one fungal cell) was counted, and the phagocytic index (number of fungal cells taken up per 100 macrophages) was calculated. (B) The cells incubated with or without DOPA were suspended in PBS, and 1.0 × 10^7^ cells were exposed to UV at 30 000 μJ·cm^−2^ for 30 s or treated with 1.5 mm H_2_O_2_ for 2 h; surviving cells were spread onto the YPD plate for colony growth, and the viability was further calculated. (C) The DOPA‐incubated cells were treated with 32 μg·mL^−1^ caspofungin or 1 μg·mL^−1^ AMB for 2 h and then spread onto the YPD plate for survival analysis. Bars, mean ± SD. Data are representative of at least three independent experiments, each with similar results. *P < 0.05, **P < 0.01, by Student's *t*‐test. ns, no significance; V, vector.

Melanin is an efficient free radical scavenger [49, 50] and confers resistance to UV light by absorbing a broad spectrum of electromagnetic waves, thus preventing photo‐induced damage. To investigate the functional role of melanin products in protecting *C. albicans* against damage, we first performed a BMDM macrophage phagocytosis assay. As shown in Fig. 9A, the DOPA preincubated *C. albicans* cells survived better than the non‐DOPA‐treated cells and showed a lower phagocytosis ratio and phagocytosis index. These results suggest that melanized *C. albicans* cells are resistant to BMDM phagocytosis and protect themselves against host immune killing. Next, we tested the melanization of *C. albicans* on other antistress responses and found that after 30 s of treatment with 30 000 μJ·cm^−2^ UV, approximately 10% of the melanized cells survived, whereas those nonmelanized cells almost all died (Fig. 9B, left). After 2 h of incubation with 1.5 mm H_2_O_2_, approximately 60% of the DOPA‐pretreated cells survived, but only 4% of the non‐DOPA‐treated cells survived (Fig. 9B, right). The DOPA‐pretreated cells were also more resistant to the antifungal drugs. Approximately 60% of the DOPA‐pretreated cells survived when they were incubated with caspofungin (32 μg·mL^−1^) or AMB (1 μg·mL^−1^) for 2 h, whereas few non‐DOPA‐treated cells survived (Fig. 9C). To rule out the possibility that the DOPA‐pretreatment itself, but not melanin production, was responsible for the protective effects observed, we included the *5fetsΔ/Δ* mutant as controls. Compared with the WT cells, the melanin production‐defective mutant cells were equally sensitive to all the damage with or without DOPA pretreatment (Fig. 9). Therefore, melanin production with exogenous DOPA substrate protected *C. albicans* cells against BMDM macrophage phagocytosis, UV radiation, H_2_O_2_ oxidation and antifungal drugs.

### Fets are important for *C. albicans* against oxidant attacks

**Fig. 10:**
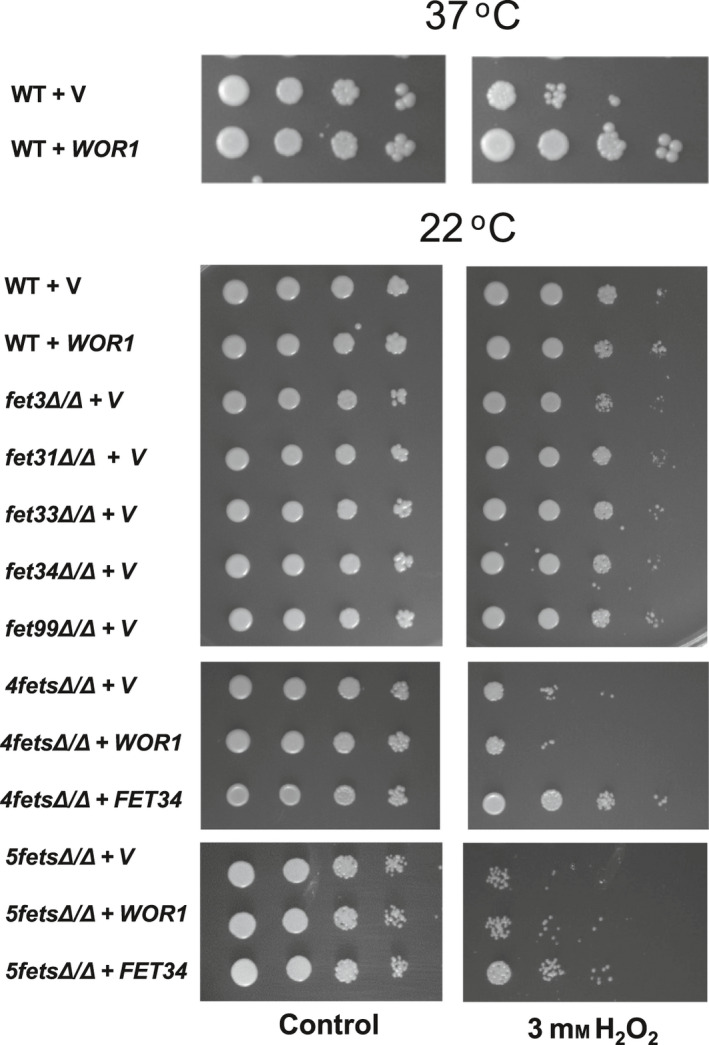
Susceptibility of *C. albicans* to H_2_O_2_. The overnight cultures of *C. albicans* cells were serially diluted by 10‐fold, spotted onto SCD plates containing 20 μm FeCl_3_ with or without 3 mm H_2_O_2_, and incubated at 22 or 37 °C for 3 days. Strains WT (JYC5) (***a/a***), *fet3Δ/Δ* (CDB2) (*ɑ/ɑ*), *fet31Δ/Δ* (CDB4) (***a/a***), *fet33Δ/Δ* (CDB5) (***a/a***), *fet34Δ/Δ* (CDB6) (***a/a***), *fet99Δ/Δ* (CDB7) (***a/a***), *4fetsΔ/Δ* (CDB9) (***a/a***) and *5fetsΔ/Δ* (CDB11) (***a/a***) were introduced with a vector (pBA1) or *WOR1* (pACT1‐WOR1). The *fet34Δ/Δ* (CDB6) (***a/a***) carried the vector pPR671. The pBA1‐FET34 was introduced into the *4fetsΔ/Δ* or *5fetsΔ/Δ* mutant for complementation.

All five *C. albicans* Fets contained three multicopper binding domains that were conserved in MCOs, such as human ceruloplasmin (HsCp). HsCP has antioxidant capacity and plays multiple functions during the life process [51]. To examine the antioxidant capacity of the *C. albicans* Fets, we spotted Fets mutant cells onto H_2_O_2_‐containing plates. At the human physiological temperature of 37 °C, cells overexpressing Wor1 grew better than the WT cells on plates containing 3 mm H_2_O_2_ (Fig. 10, upper panels). At an opaque maintaining temperature of 22 °C *in vitro*, the growth of opaque cells overexpressing Wor1 was similar to that of WT white cells (Fig. 10, lower panels). To eliminate the effect of ferric ions on cell growth, all plates were supplied with 20 μm FeCl_3_ [29]. All the single *fet* gene deletion mutants had no growth defect on the H_2_O_2_‐containing plates at 22 °C, but *4fetsΔ/Δ* and *5fetsΔ/Δ* mutant cells were sensitive to H_2_O_2_ (Fig. 10, lower panel). Given the slow growth rate of the *4fetsΔ/Δ* and *5fetsΔ/Δ* mutant cells, we incubated them on the plate for a longer time. Wor1 overexpression in the *4fetsΔ/Δ* or *5fetsΔ/Δ* mutant could not recover cell growth in the medium containing H_2_O_2_, but reintroducing *FET34* from the *ADH1* promoter into the *4fetsΔ/Δ* or *5fetsΔ/Δ* mutant restored cell growth. All these findings suggest that the *C. albicans* MCO Fets have antioxidant capacity.

## Discussion

**Fig. 11:**
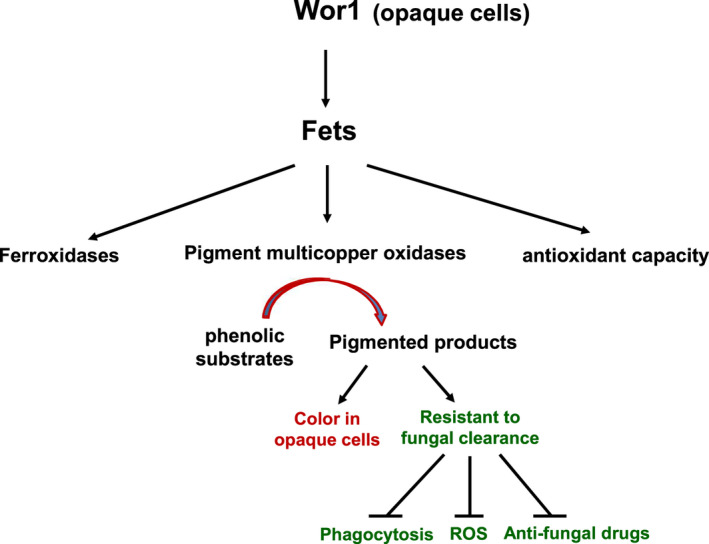
Wor1‐up‐regulated Fets function as pigment MCOs required for pigment formation in opaque cells and contribute to antioxidant reaction. *C. albicans* presents as a commensal resident in the human gut, which is thought to be replete in GlcNAc, and with high CO_2_. *WOR1* overexpression promotes white‐to‐opaque or white‐to‐GUT switching. Wor1 up‐regulates the *FETs* expression in rich media at the physiological temperature of 37 °C or in glucose minimal media with LA. The increased Fets function as pigment MCOs to promote melanin production using exogenous DOPA or other phenolic substrates. At low iron host niches, the up‐regulated Fets function as Fets to facilitate iron acquisition. The Fets also have antioxidant capacity to protect the *C. albicans* against oxidative damage. The distinct expression patterns of the *FET* genes and the diverse functions of the Fets reflect the ability of *C. albicans* to rapidly adapt to diverse host niches and survive in the host.

Previous studies [30, 31] have shown that *C. albicans* can produce dark‐pigmented melanin *in vitro* and during infection. However, no gene encoding laccase has been identified thus far. Here, we demonstrated that the MCO Fets, which were previously reported as Fets [29], function as pigment MCOs and promote melanin production under the control of Wor1 in opaque cells (Fig. 11). *C. albicans* can undergo white‐opaque switching, and the white and opaque cells have known distinct features. We found that the dark color of opaque colonies was associated with increased *FETs* expression and melanin production, and the pimpled surface of opaque cells was associated with melanin externalization.

We also found evidence that Wor1 had additional regulatory impacts on the expression of *FETs*, along with iron. The five *FET* genes had distinct expression patterns in response to iron concentrations, among which *FET34* and *FET99* were down‐regulated by high iron concentrations and up‐regulated by low iron concentrations, *FET31* was slightly up‐regulated by low iron concentrations, and *FET3* and *FET33* exhibited iron‐independent expression [29, 34]. To examine the impact of Wor1 on the *FET* gene transcription, we cultured *MTL* homozygous *WOR1‐*stabilized opaque cells (WT + *WOR1*) and WT white cells (WT + vector) in rich or poor medium under different growth conditions. In YPD‐rich medium, Wor1 overexpression greatly up‐regulated four (*FET3*, *FET31*, *FET33* and *FET34)* of the five *FET* genes, especially at the host physiological temperature of 37 °C, but had a subtle effect on *FET99* expression. In GMM, all five *FET* genes were up‐regulated by Wor1 with LA. However, *FET99* was down‐regulated by Wor1 in minimal medium with high ammonium. In addition, high CO_2_ combined with high concentrations of GlcNAc at 37 °C mimicking the host GI tract environment also up‐regulated *FET31* and *FET34* expression. As investigated previously, genome‐wide gene expression comparisons showed different *FET* gene expression patterns in white and opaque cells [29, 52, 53, 54, 55]. Because different media and growth conditions were used in each report, we speculated on whether the components in the media and growth conditions had any impact on the regulation of *FET* genes. Therefore, the expression of *FET* genes is controlled in multiple layers in response to different environmental stimuli, which reflect the multiple enzymatic activities of Fets in different host niches.

The host GI tract is a complex and changeable environment where large numbers of microorganisms live inside. Knowing that trillions of microorganisms reside in the human GI tract, the gut microbiota is now regarded as a virtual endocrine organ that produces abundant chemicals of hormonal nature, such as the precursor to neuroactive compounds l‐DOPA [56]. Therefore, it is especially important for commensal pathogen *C. albicans* to rapidly adapt to diverse host niches, furnishing iron and other chemicals in different forms and levels. Under iron‐limited conditions, the Fets Fet34 and Fet99 were up‐regulated and partnered with membrane permeases to form a high‐affinity iron transporter to acquire iron. In the GI tract, which is thought to be replete in GlcNAc, and with high CO_2_ [7, 48], the up‐regulated Fets function as pigment MCOs to catalyze the exogenous l‐DOPA or other phenolic substrates to form melanin pigment. Given that *C. albicans* is a commensal resident in the human gut, *C. albicans* can undergo white‐to‐opaque switching in host gut conditions [7, 47, 48], and Wor1 overexpression triggers white‐to‐GUT switching and promotes *C. albicans* commensalism in the mouse gut [3]. Our finding that Wor1 up‐regulated Fets and subsequently catalyzed melanin production may explain the different appearances between the opaque and GUT forms. Wor1 can promote white‐to‐opaque switching in *MTL* homozygous cells and trigger white‐to‐GUT switching in *MTL* ***a*** */α* heterozygous cells. According to our data in Fig. 2, the melanin production in *MTL* homozygous cells overexpressing Wor1 was higher than that in *MTL* ***a*** */α* heterozygous cells overexpressing Wor1. We speculate that the pimpled surface of the opaque form in *MTL* homozygous cells may be associated with the exhaustion of melanin externalization, and that the smooth surface of GUT form in *MTL* ***a*** */α* heterozygous cells is probably associated with less melanin secretion.

Melanins are used by microbes to protect pathogens against host immune responses [57], and conversely used by the host to defend against microbes [58]. Fungal melanin also can protect microbes against oxidants, UV, heat, enzymatic degradation, and antimicrobial compound stress [50]. DOPA‐melanin plays an immunomodulatory role in *C. neoformans* infection [59, 60] by inhibiting host cell phagocytosis and cytokine production and reducing the toxicity of microbicidal peptides. DHN‐melanin from *Aspergillus fumigatus* facilitates the microbes to inhibit host cell apoptosis [61] and spread within the host niche [62]. DHN‐melanin can also be sensed by the host through a melanin‐sensing C‐type lectin receptor (MelLec), which plays a crucial role in the control of systemic *A. fumigatus* infection in both mice and humans [63]. The *C. albicans‐*produced melanin not only contributes to the color formation of opaque cells but also plays a protective role in *C. albicans* infection. Melanized *C. albicans* cells are more resistant to BMDM phagocytosis, radiation and oxidative damage and are tolerant to antifungal drugs.

In addition, we found that Fet MCOs had antioxidant capacity and functionally resembled the mammalian ceruloplasmin. Ceruloplasmin is a blue‐colored plasma protein with multiple physiological functions, including copper transport, oxidation of organic amines, radical scavenging, Fet activity and antioxidant activity [28, 51]. Ceruloplasmin inhibits the ferrous ion‐dependent formation of hydroxyl radicals in the Fenton reaction, scavenges ROS and protects hepatocytes from oxidative damage in orthotopic liver transplantation [51]. *C. albicans* acquires iron from specific host molecules in regions where iron is scarce, while also defending against iron overdose toxicity in regions where iron occurs in excess [11, 64]. Therefore, *C. albicans* possesses five Fets representing the functional diversity of the enzymes, as Fets required for iron acquisition and laccases/pigment MCOs required for melanin production and antioxidant ability, befitting its commensal‐pathogenic lifestyle.

## Conflict of interest

The authors declare no conflict of interest.

## Author contributions

BD and JC designed the project. BD, YX and NG performed experiments. BD, YX and JC analyzed data. BD and JC wrote the manuscript. All of the authors read and approved the final manuscript.

## Supporting information


**Fig. S1.** PCR and Southern blotting for *FET* gene deletion. (A) PCR analysis of deletion of *FET31 (orf19.4211)* in *Candida albicans* as a representative. PAP: LoxP‐CdARG4‐LoxP; PHP: LoxP‐CdHIS1‐LoxP. P1: AGCCTCCTCCTCATCATCTT; P2: ATTTGAACGGACTGCACATA; P3: AACACACCATCGAAAAAGTCG; P4: CAACCTTTCAAACGATGCAA; P5: CATTTCACACCCAGCTCGTA; P6: ACGACGGCTGATTTGTCTTT. (B) Southern blotting of *FET3 (orf19.4213)* deletion. The genomic DNA digested with KpnI. Probe FET3‐F: GATGAGACATGAGAGGAAGCTATT, Probe FET3‐R: CCGAACCCTGTTGTTGTAGT. (C) Southern blotting of *FET99 (orf19.4212)* deletion. The genomic DNA digested with XbaI. Probe FET99‐F: CATCAGGTAGGCTTAGAAGA, Probe FET99‐R: AATAGTTGATTAGCCGTTGC. Biotin‐labeled (North2South™ Biotin Random Prime DNA Labeling Kit, Thermo).

**Fig. S2.** Total proteins were estimated by the Coomassie brilliant blue method.

**Fig. S3.** Representative TEM of *Candida albicans* cells. JYC5 + pACT1‐WOR1 opaque cells cultured with no addition of DOPA (A) or with 1 mm DOPA (B) at 22 °C for 4 days. Bars, 0.5 μm.

**Fig. S4.** Melanin production in SC5314 and CAI4 + V cells. Melanin production by *MTLa/a* SC5314 and CAI4 + V cells were determined by optical density at 310 nm (OD_310_) or 470 nm (OD_470_) for whole cultures (A, C) and in resuspended cell pellets (B, D). (A, B) For white cells; (C, D) for opaque cells. Melanin production by *MTL a/α* SC5314 and CAI4 + V white cells for whole cultures (E) and in resuspended cell pellets (F). Data represent the mean ± SD (*n* ≥ 3 independent experiments).

**Fig. S5.** Melanin‐associated oxidase activity in *MTLa/a* SC5314 and CAI4 + V (JYC5 + V) white cells. The white cells were cultured in YPD at 22 or 37 °C and collected for extraction of total proteins. Specific oxidase activity was determined using DOPA as a substrate and indicated as mU Abs·min^−1^·μg protein^−1^. Bars, mean ± SD. Data are representative of at least three independent experiments, each with similar results. Data are relative to OD_310_ in SC5314 cells. ns, no significance, by Student's *t*‐test.

**Fig. S6.** Prediction of Wor1 binding sites on the promoters according to previous reports.

**Fig. S7.** Mating efficiency of the *MTLa 5fets* mutant. The tester WT *α* strain used is CHY477 (*mtla1/MTLα URA3 HIS1 ade2*). JYC5 is WT *MTLa ura3 ADE2* strain. CBD11 is *5fets MTLa ura3 ADE2* mutant. Mating efficiency = mean ± SD. Data relative to mating efficiency in the WT cell. *P*_White_ = 0.85, *P*_Opaque_ = 0.52, by Student's *t*‐test. ns, no significance.

## Data Availability

The authors declare that the main data supporting the findings of this study are available within the article and its Supplementary Information. Extra data that support the findings of this study are available from the corresponding authors upon reasonable request.
