# Supplementary figures and images for: Wor1‐regulated ferroxidases contribute to pigment formation in opaque cells of Candida albicans
Source: FEBS Open Bio. 2021 Feb 18;11(3):598–621. doi: 10.1002/2211-5463.13070 (PMC7931227; doi:10.1002/2211-5463.13070)

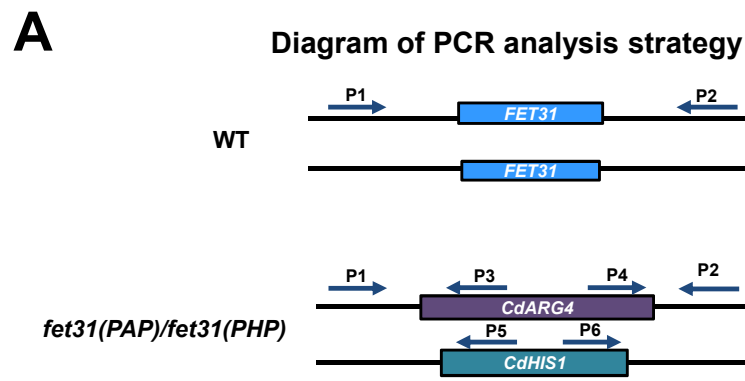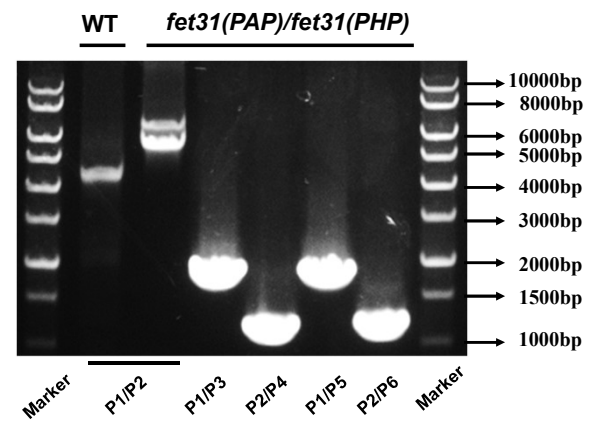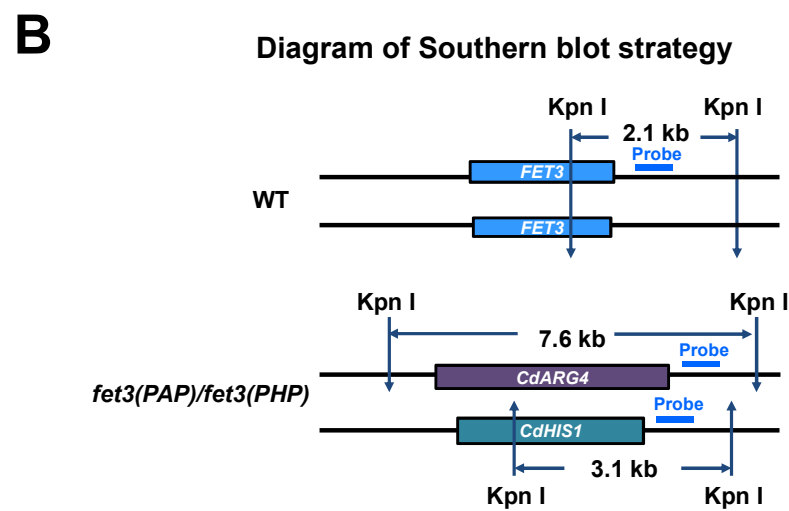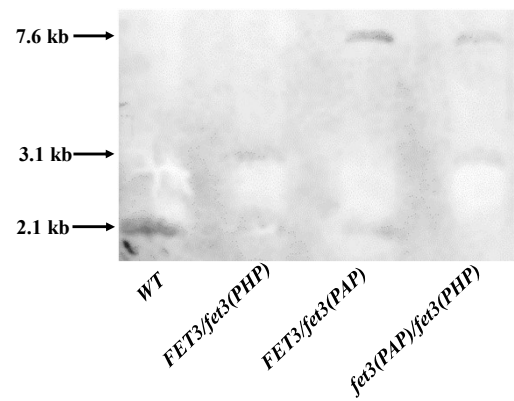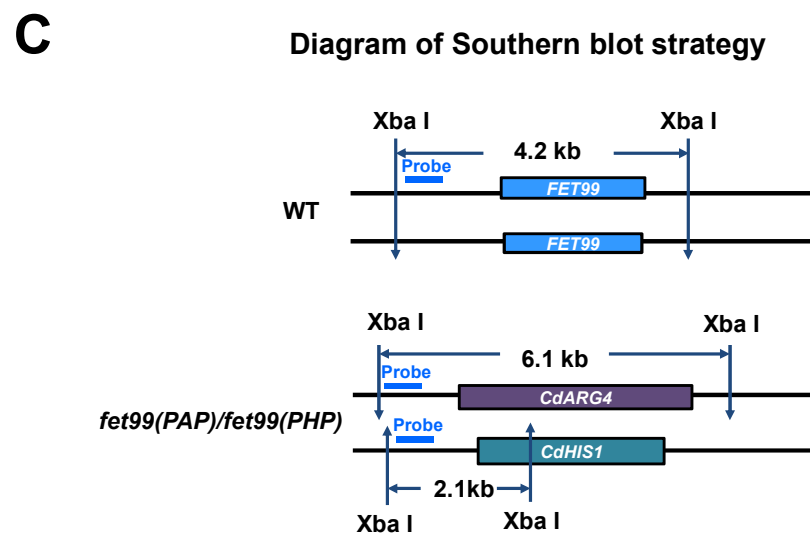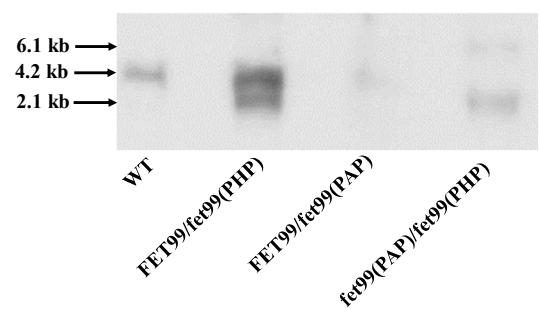

Supplementary Figure 1

Supplement: Supplementary file 1 — Fig. S1. PCR and Southern blotting for FET gene deletion. (A) PCR analysis of deletion of FET31 (orf19.4211) in Candida albicans as a representative. PAP: LoxP‐CdARG4‐LoxP; PHP: LoxP‐CdHIS1‐LoxP. P1: AGCCTCCTCCTCATCATCTT; P2: ATTTGAACGGACTGCACATA; P3: AACACACCATCGAAAAAGTCG; P4: CAACCTTTCAAACGATGCAA; P5: CATTTCACACCCAGCTCGTA; P6: ACGACGGCTGATTTGTCTTT. (B) Southern blotting of FET3 (orf19.4213) deletion. The genomic DNA digested with KpnI. Probe FET3‐F: GATGAGACATGAGAGGAAGCTATT, Probe FET3‐R: CCGAACCCTGTTGTTGTAGT. (C) Southern blotting of FET99 (orf19.4212) deletion. The genomic DNA digested with XbaI. Probe FET99‐F: CATCAGGTAGGCTTAGAAGA, Probe FET99‐R: AATAGTTGATTAGCCGTTGC. Biotin‐labeled (North2South™ Biotin Random Prime DNA Labeling Kit, Thermo). [file FEB4-11-598-s001.pdf]

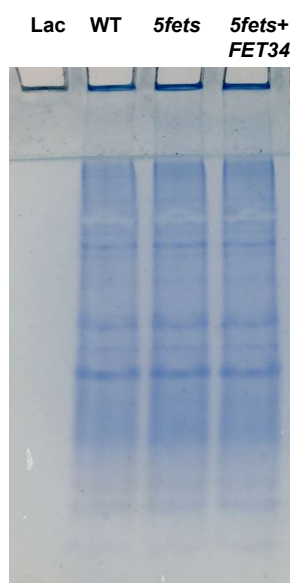

Supplementary Figure 2

Supplement: Supplementary file 2 — Fig. S2. Total proteins were estimated by the Coomassie brilliant blue method. [file FEB4-11-598-s002.pdf]

**A**

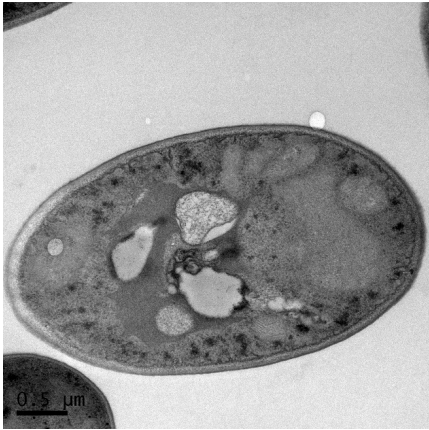

**B**

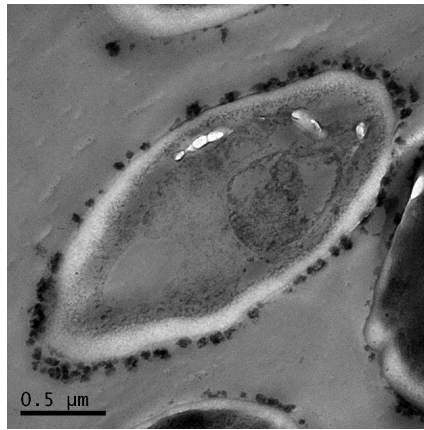

Supplementary Figure 3

Supplement: Supplementary file 3 — Fig. S3. Representative TEM of Candida albicans cells. JYC5 + pACT1‐WOR1 opaque cells cultured with no addition of DOPA (A) or with 1 mm DOPA (B) at 22 °C for 4 days. Bars, 0.5 μm. [file FEB4-11-598-s003.pdf]

## A *MTLa/a* White

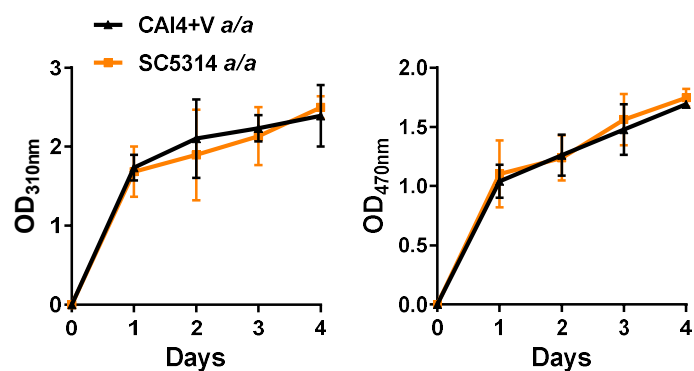

## B

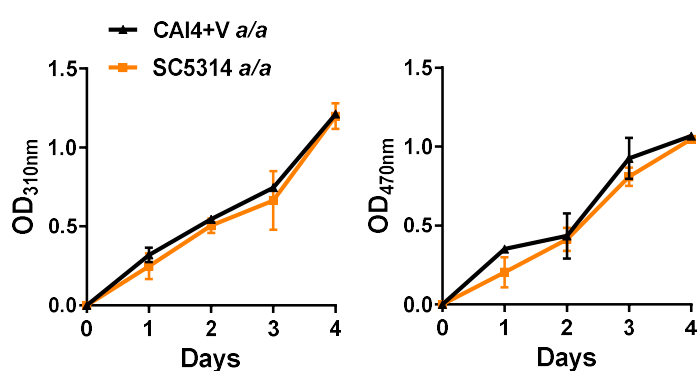

## C *MTLa/a* Opaque

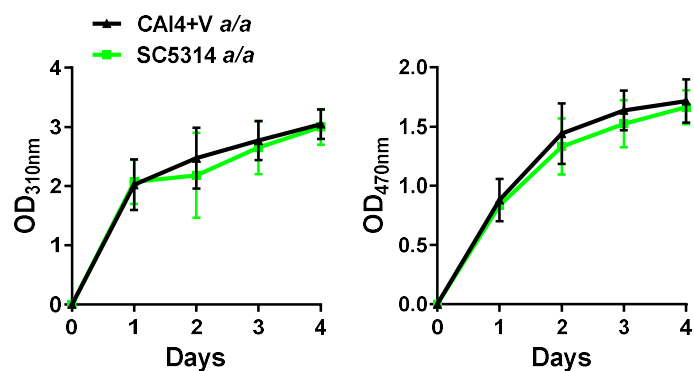

## D

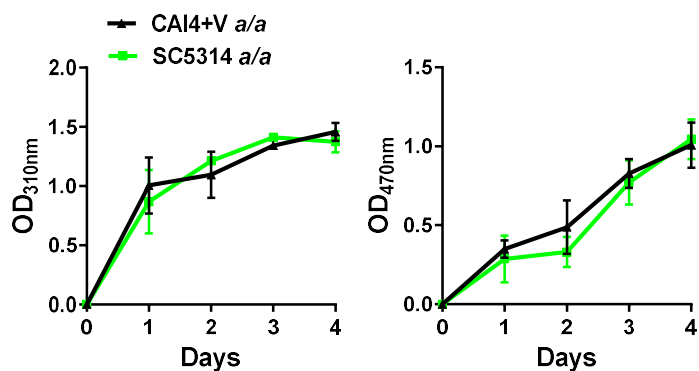

## E *MTLa/α*

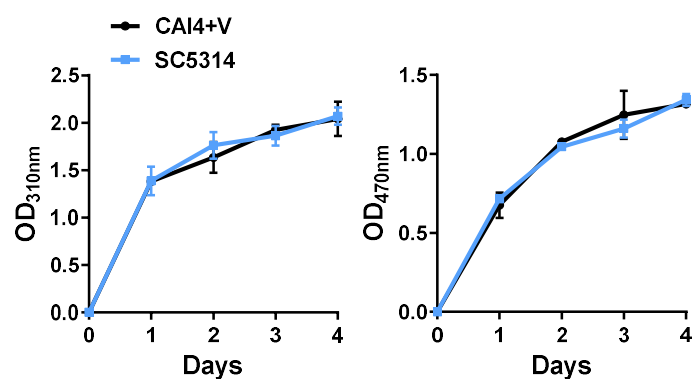

## F

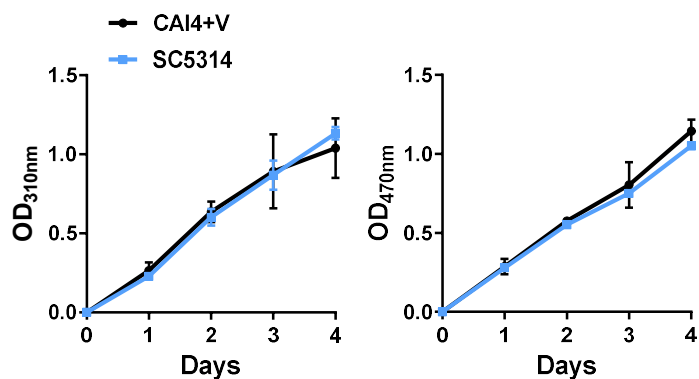

Supplementary Figure 4

Supplement: Supplementary file 4 — Fig. S4. Melanin production in SC5314 and CAI4 + V cells. Melanin production by MTLa/a SC5314 and CAI4 + V cells were determined by optical density at 310 nm (OD310) or 470 nm (OD470) for whole cultures (A, C) and in resuspended cell pellets (B, D). (A, B) For white cells; (C, D) for opaque cells. Melanin production by MTL a/α SC5314 and CAI4 + V white cells for whole cultures (E) and in resuspended cell pellets (F). Data represent the mean ± SD (n ≥ 3 independent experiments). [file FEB4-11-598-s004.pdf]

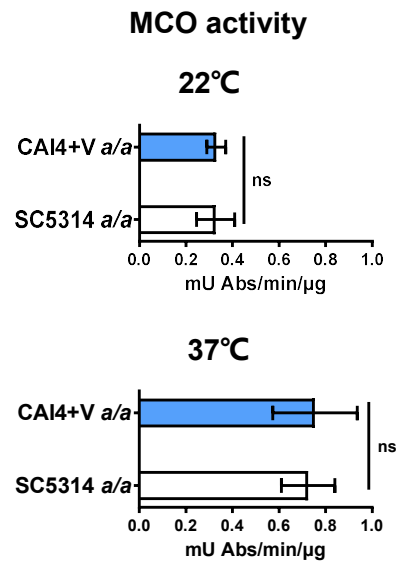

Supplementary Figure 5

Supplement: Supplementary file 5 — Fig. S5. Melanin‐associated oxidase activity in MTLa/a SC5314 and CAI4 + V (JYC5 + V) white cells. The white cells were cultured in YPD at 22 or 37 °C and collected for extraction of total proteins. Specific oxidase activity was determined using DOPA as a substrate and indicated as mU Abs·min−1·μg protein−1. Bars, mean ± SD. Data are representative of at least three independent experiments, each with similar results. Data are relative to OD310 in SC5314 cells. ns, no significance, by Student's t‐test. [file FEB4-11-598-s005.pdf]
