# Supplementary material for: Wor1‐regulated ferroxidases contribute to pigment formation in opaque cells of Candida albicans
Source: FEBS Open Bio. 2021 Feb 18;11(3):598–621. doi: 10.1002/2211-5463.13070 (PMC7931227; doi:10.1002/2211-5463.13070)
Supplement: Supplementary file 6 — Fig. S6. Prediction of Wor1 binding sites on the promoters according to previous reports. [file FEB4-11-598-s006.pdf]

Genes	Wor1 binding sites
FET3(orf19.4213)	-1407 ACTGAATATTCTTTTGTTGC -1388
	-1294 GACAATTACCCTTTTTAACC -1275
FET31(orf19.4211)	-3830 CATTTATATAGTTTCCCTTG -3811
	-2327 TGATTTTAATGTTTTGTTAA -2308
	-2187 GCCACATAGACTTTAAAATA -2168
FET33(orf19.943)	-912 TATAGGTACAGTTTATCAAG -893
FET34(orf19.4215)	-1295 ACTTTTTAGACTTTTTAAAT -1276
FET99(orf19.4212)	-907 TCCCGCTATTGTTTTCTCTG -888

Genes	Wor1 binding sites
FTR1(orf19.7219)	-164 TTAGAGTATTGTTTTTTTGG -145
FTR2(orf19.7231)	-211 TTCTAGTATTCTTTTCTTAC -192
FTH1(orf19.4802)	-438 TAGTGTTAGAGTTTTGTTAA -419
FTH2(orf19.3227)	-1369 TTCTCTTACTCTTTTCTTCA -1350
WOR1(orf19.4884) site 1	-6039 GTTAAAAAACTCTATTTTCA -6020
WOR1(orf19.4884) site 2	-5989 AAGAAGTTAAACTTTTTTGA -5970
WOR1(orf19.4884) site 3	-5849 GGAATAATTAGAGTTTTACA -5830

Supplementary Figure 6
