# Supplementary material for: Wor1‐regulated ferroxidases contribute to pigment formation in opaque cells of Candida albicans
Source: FEBS Open Bio. 2021 Feb 18;11(3):598–621. doi: 10.1002/2211-5463.13070 (PMC7931227; doi:10.1002/2211-5463.13070)
Supplement: Supplementary file 7 — Fig. S7. Mating efficiency of the MTLa 5fets mutant. The tester WT α strain used is CHY477 (mtla1/MTLα URA3 HIS1 ade2). JYC5 is WT MTLa ura3 ADE2 strain. CBD11 is 5fets MTLa ura3 ADE2 mutant. Mating efficiency = mean ± SD. Data relative to mating efficiency in the WT cell. P White = 0.85, P Opaque = 0.52, by Student's t‐test. ns, no significance. [file FEB4-11-598-s007.pdf]

	Mating efficiency	
a strain/White	× α White	× α Opaque
WT	$(7.1 \pm 2.3) \times 10^{-7}$	$(1.9 \pm 0.5) \times 10^{-6}$
5fetsΔ/Δ	$(8.5 \pm 1.3) \times 10^{-7\ ns}$	$(1.8 \pm 0.2) \times 10^{-6\ ns}$

Supplementary Figure 7
